# Transcriptome Analysis Reveals the Important Role of *WRKY28* in *Fusarium oxysporum* Resistance

**DOI:** 10.3389/fpls.2021.720679

**Published:** 2021-08-20

**Authors:** Jian Diao, Jiaqi Wang, Ping Zhang, Xin Hao, Yang Wang, Liwei Liang, Yue Zhang, Wei Ma, Ling Ma

**Affiliations:** ^1^Forest Protection, College of Forestry, Northeast Forestry University, Harbin, China; ^2^Medicinal Plant, College of Medicine, Heilongjiang University of Chinese Medicine, Harbin, China

**Keywords:** *Fusarium oxysporum* tolerance, RNA-seq, pathogen-plant interaction, gene overexpression, genetic transformation, transcription factor

## Abstract

Root rot of *Populus davidiana* × *P. alba* var. *pyramidalis* Louche (Pdpap) is caused by *Fusarium oxysporum*. We used RNA sequencing to study the molecular mechanisms and response pattern of Pdpap infected by *F. oxysporum* CFCC86068. We cloned the *PdpapWRKY28* transcription factor gene and transformed the recombinant vector pBI121-*PdpapWRKY28* into Pdpap. The resistance function of *PdpapWRKY28* was verified using physiological and biochemical methods. By means of RNA sequencing, we detected 1,403 differentially expressed genes (DEGs) that are common in the different treatments by *F. oxysporum*. Furthermore, we found that overexpression of the *PdpapWRKY28* gene may significantly improve the resistance of Pdpap plants to *F. oxysporum*. Our research reveals a key role for *PdpapWRKY28* in the resistance response of Pdpap to *F. oxysporum*. Additionally, our results provide a theoretical basis for in-depth research on resistance breeding to combat root rot.

## Introduction

*Populus davidiana* × *P. alba* var. *pyramidalis* Louche (Pdpap), as an important greening tree species, has extremely high production and consumption values (Yu and Zhang, 2005; Guo, 2019). Recently, a large-scale outbreak of root rot caused by *Fusarium oxysporum* has severely hindered the growth and development of Pdpap. Studies have shown that root rot causes radiation-induced disease in plants in a short span of time (Elad and Pertot, 2014). Its pathogenic mechanism has three main approaches: blocking plant ducts (King et al., 2011), secreting toxic substances (Wang et al., 2000; Bani et al., 2014), and expressing pathogenic genes (Ma et al., 2013; Schmidt et al., 2013). Presently, the prevention and control of root rot mostly rely on physical or chemical methods (Duan et al., 2010; Zhang et al., 2012; Tu et al., 2014; Pei et al., 2016). However, pesticide use and physical defenses have limited effects on root rot control and treatment. Therefore, we considered directly studying Pdpap and exploring their responses to *F. oxysporum*. Finally, in the event of finding or inducing favorable outcomes, we made sure that disease-resistant breeding should be realizable.

Few studies on the response mechanism of Pdpap to *F. oxysporum* have been conducted, but the functional exploration of plant genes responding to pathogens has been extensive. Studies have shown that the response of Pdpap to a wide range of pathogens was regulated by multiple proteins; among them, transcription factors were closely related to the stress response of the plant (Sakuma et al., 2002). The process by which transcription factors enhance the ability of a plant to resist stress is mainly by increasing the expression level of transcription factors (Ali et al., 2013), which can further regulate the expression of functional downstream genes (Stein et al., 2001; Hou et al., 2009). The WRKY transcription factor was the first transcriptional regulatory factor found in plants (Rushton et al., 2010). It has been isolated from multiple species and was found to play an important regulatory role in plant signal transduction and response to biotic and abiotic stresses (Wei et al., 2012). Studies showed that *PtWRKY28* belongs to the subgroup IIb of WRKY proteins (He et al., 2015; Imran et al., 2019) and participates in the regulatory process of plant resistance to pathogens (He et al., 2015). For example, *AtWRKY28* is involved in the resistance response of *Arabidopsis* to the necrotrophic pathogens *Botrytis cinerea* (Wu et al., 2011) and *Sclerotinia sclerotiorum* and oxalic acid. Babitha et al. (2013) reported that co-expression of *AtWRKY28* and *AtbHLH17* in *Arabidopsis* conferred resistance to abiotic stress such as that produced by sodium chloride (NaCl), polyethylene glycol, and methyl viologen. Chujo et al. (2013) reported that *OsWRKY28* overexpression negatively regulated the innate immune responses of rice against rice blast fungus, resulting in reduced resistance. Wang et al. (2020) reported that overexpression of *SlWRKY28* could improve the tolerance of *P. davidiana* × *P. bolleana* to alkaline salt stress.

In this study, we performed RNA sequencing in wild type (WT) Pdpap and Pdpap treated with *F. oxysporum* CFCC86068. The results of the RNA sequencing indicated that *PdpapWRKY28* may play an important role in the response of Pdpap to *F. oxysporum*. We cloned the 977 bp cDNA fragment of the *PdpapWRKY28* gene from Pdpap. We constructed the recombinant vector pBI121-*PdpapWRKY28* for the overexpression of *PdpapWRKY28* under the control of the cauliflower mosaic virus (CaMV) 35S promoter. The transcription factor and its promotor were successfully transformed into Pdpap. In addition, evidence based on the analysis of molecular, morphological, and physiological data showed that the *PdpapWRKY28*-overexpression transformants enhanced resistance to *F. oxysporum* CFCC86068. The results showed that *PdpapWRKY28* had a positive response to root rot, which strongly suggests that *PdpapWRKY28* could be used as an effective target for breeding resistance to root rot.

## Materials and Methods

### Sample Preparation

The WT Pdpap used in this experiment was provided by the Forest Protection Department of the Forestry College of Northeast Forestry University (Harbin, Heilongjiang, China). WT Pdpap seedlings were cultured on 0.5X Murashige and Skoog (MS) medium supplemented with 0.01 mg/ml of 1-naphthaleneacetic acid (NAA). For stable gene transformation, leaves from 1-month-old Pdpap were transferred to a differentiation medium that contained 0.5 mg/ml of 6-benzylaminopurine, 0.1 mg/ml of NAA, and 0.02 mg/ml of Thidiazuron. Tissue-cultured Pdpap was grown in a plant tissue culture room. In order to simulate Pdpap growth under natural conditions, plants at the same growth stage were selected, transplanted into artificial soil, and cultivated in an artificial climate room at a temperature of 22 ± 2°C with a light intensity of 400 lx/m^2^/s, a photoperiod of 16 h of light and 8 h of dark, and a relative humidity of 65–75% (Lu et al., 2011).

*Fusarium oxysporum* CFCC86068 was obtained from the China Forestry Culture Collection Center (Beijing, China). The preactivated *F. oxysporum* was transferred to a potato dextrose agar (PDA) medium and cultured in the dark for 7 days. We used a 9-mm-diameter cork borer to punch holes at the edge of the hyphae and then transferred the punched-out disks into a new PDA medium. After culturing in the incubator at 28°C for 14 days, the spores were washed five times with sterile water. After washing, spores were filtered through eight layers of gauze and collected into a 50-ml centrifuge tube. Spore density calculated using a hemocytometer was about 1 × 10^5^/ml. The spore-bearing medium was then sealed and stored at room temperature for later use. Spores were recultured each day at a sterile workbench.

Two-month-old WT Pdpap plants at the same growth stage were selected. Fifty milliliters of *F. oxysporum* at a spore density of 1 × 10^5^/ml was poured onto Pdpap roots (Kang et al., 2018). Infection times were 0, 6, 12, 24, or 48 h. The WT Pdpap plants treated with 50 ml of ddH_2_O were used as control. Every treatment consisted of duplicated samples. Afterward, all samples were immediately transferred to liquid nitrogen for RNA sequencing analysis.

### RNA Sequencing

The RNAprep Pure Plant Plus Kit (Tiangen, Beijing, China) protocol was used to extract the total RNA of 20 samples. Three micrograms of RNA per sample were used as input for RNA sample preparation. Sequencing libraries were generated using the NEBNext^®^ UltraTM RNA Library Prep Kit for Illumina (NEB, Beijing, China) following the recommendations of the manufacturer. Library quality was assessed on an Agilent Bioanalyzer 2100 system (Agilent, Santa Clara, CA, USA). After quality inspection, the libraries were pooled according to effective concentration and quantity (Ihle et al., 2014). The library preparations were sequenced on the Illumina HiSeqTM 2500 (Illumina, San Diego, CA, USA) and 125 bp/150 bp paired-end reads were generated. RNA extraction, RNA-seq library construction, and sequencing were performed by Novogene (Beijing, China). The clean reads can be found in the National Center for Biotechnology Information (NCBI) Sequence Read Archive (SRA) repository (https://submit.ncbi.nlm.nih.gov/subs/sra/), and the accession number is PRJNA741264.

### Sequencing Data Analysis

Raw data (raw reads) in the FASTQ format were first processed through in-house Perl scripts (Andrews, 2010). In this step, clean data (clean reads) were obtained by removing reads containing adapters, poly-N sequences, and low-quality reads from the raw data. The Q20, Q30, and guanine-cytosine (GC) content of the clean data were calculated. All downstream analyses were based on the clean data. Reference genome and gene model annotation files were downloaded directly from the genome website (https://www.ncbi.nlm.nih.gov/genome/?term=populus$+$trichocarpa). The index of the reference genome was built using Hisat2 (v2.0.5) (Kim et al., 2015) and paired-end clean reads were aligned to the reference genome using the same software. The mapped reads of each sample were assembled using StringTie (v1.3.3b) (Pertea et al., 2015) in a reference-based approach. StringTie uses a novel network flow algorithm and an optional *de novo* assembly step to assemble and quantitate full-length transcripts representing multiple splice variants for each gene locus. FeatureCounts (v1.5.0-p3) (Liao et al., 2014) was used to count the number of reads mapped to each gene. Then, the fragments per kilobase million (FPKM) for each gene was calculated based on the length of the gene and the read count mapped to the gene. The FPKM refers to the expected number of fragments per kilobase of transcript sequenced (length) per million base pairs sequenced (depth). Differential expression analysis of the two conditions/groups (two biological replicates per condition) was performed using the DESeq2 R package (1.16.1) (Love et al., 2014). DESeq2 provides statistical routines for determining differential expression in digital gene expression data using a model based on the negative binomial distribution. The resulting *P*-values were adjusted using the approach by Benjamini and Hochberg for controlling the false discovery rate (Madar and Batista, 2016). The genes with an adjusted *P-*value < 0.05 found by DESeq2 were considered to be differentially expressed. Gene ontology (GO) (Young et al., 2010) enrichment analysis of DEGs was implemented using the cluster profileR R package, in which gene length bias was corrected. The GO terms with corrected *P-*values < 0.05 were considered significantly enriched. The transcription factor analysis of DEGs was extracted directly from the Plant Transcription Factor Database (PlantTFDB) (https://ngdc.cncb.ac.cn/databasecommons/database/id/307) (Jin et al., 2014).

### Validation of DEGs by qRT-PCR

Expression of DEGs obtained through transcriptome sequencing was verified by quantitative real-time PCR (qRT-PCR). Total RNA was extracted from Pdpap according to the method described above. cDNA was synthesized using the PrimeScript^TM^ RT reagent Kit with gDNA Eraser (Takara, Dalian, Liaoning, China). The procedures used were described in the instructions for the kits. We randomly selected five upregulated and five downregulated DEGs, for which the cDNA molecules were used as templates and verified by qRT-PCR (Xu et al., 2021; Yang et al., 2021). Their specific primers were designed using Primer 5.0 (Supplementary Table 1) (Zhai et al., 2008). *Pdpapactin* and *PdpapEF1-*α were used as internal control genes (Supplementary Table 1) (Jiang, 2017). Quantitative RT-PCR was performed on a Stratagene Mx3000P real-time PCR system (Agilent Technologies, Santa Clara, CA, USA) using the 2 × SYBR Green qPCR Master Mix kit (Bimake, Shanghai, China) according to the instructions of the manufacturer. The amplification curve was generated after analyzing the raw data. The cycle threshold (Ct) value was calculated based on a fluorescence threshold of 0.01 (Wang et al., 2012). The reaction systems of qRT-PCR are shown in Supplementary Table 2. The qRT-PCR amplification conditions were as follows: an initial denaturation step at 94°C for 30 s followed by 44 cycles at 94°C for 12 s, 58°C for 30 s, 72°C for 45 s, and 79°C for 1 s. The reaction specificity was determined by performing a melting-curve analysis from 55 to 99°C, with fluorescence readings taken for 1 s for every 0.5°C rise in temperature. The relative expression level of target genes was calculated by 2^−ΔΔCt^ (Livak and Schmittgen, 2001), defined as ΔΔCt = (C_t−target_ – C_t−control_)_2_ – (C_t−target_ – C_t−control_)_1_. Three duplicates were made for each gene. After determining the relative expression levels of these genes, the results were compared with their FPKM value from the transcriptome.

### Screening of Genes Related to Disease Resistance and the Analysis of Expression Patterns

Based on the transcriptome, DEGs were screened according to the threshold of log_2_FoldChange > 3 and *P* adj < 0.05. According to the annotations of gene function, genes whose expression levels increased upon infection with *F. oxysporum* were selected as candidate genes for study.

To clarify the role of the candidate genes in the process of Pdpap responding to pathogen infection, we analyzed the expression patterns of the candidate genes after infecting Pdpap with *F. oxysporum* according to the method described above. The primer sequences of the candidate genes are shown in Supplementary Table 3.

### RNA Extraction and cDNA Transformation

We took 0.1 g of young leaves from WT Pdpap plants and used a TaKaRa MiniBEST Plant RNA Extraction kit (Takara Bio, Kusatsu, Shiga, Japan) to extract their total RNA. We synthesized the cDNA using the PrimeScript RT reagent Kit with gDNA Eraser (Takara Bio, Kusatsu, Shiga, Japan). The procedures adopted were described in the instructions provided with the kits.

### Evolutionary Analysis of the *PdpapWRKY28* Gene

In order to better understand the evolution of the *PdpapWRKY28* gene, 31 protein sequences thought to be closely genetically related to the *PdpapWRKY28* gene were obtained from the NCBI website (https://www.ncbi.nlm.nih.gov/) (Stoesser et al., 2014). Using the MEGA 5.1 software (Tamura et al., 2011), a phylogenetic tree was constructed using neighbor joining (NJ) clustering. When constructing the phylogenetic tree, the relevant parameters were set as follows: The construction of the NJ method used 1,000 iterations of bootstrap resampling using the Poisson model (Poisson bootstrapping). To further verify the evolutionary relationship between the proteins, we used the software program MEGA5 and the JTT (protein mutation data matrix) + F (mutation frequency data) + G (site-specific variations in mutation rate) model of evolution to build a maximum likelihood (ML) phylogenetic tree using the same sequence data.

### Differential Tissue Expression of the *PdpapWRKY28* Gene

To determine the spatial expression profiles of the *PdpapWRKY28* gene, we sampled six tissue types: roots, stems, leaves, and the upper, middle, and lower parts of stems. RNA was extracted from the tissues (each about 5 cm in length) of the 6-month-old WT Pdpap plants. The recovered RNA was subjected to reverse transcription to obtain cDNA. The expression of the *PdpapWRKY28* gene in each sample was analyzed by qRT-PCR. The qRT-PCR amplification conditions were as described above. The primer sequences used to amplify *PdpapWRKY28* are shown in Supplementary Table 3.

### Cloning of and Vector Construction for the *PdpapWRKY28* Gene

We used PCR to clone the *PdpapWRKY28* gene using the primer sequences listed in Supplementary Table 4. The components of the PCR amplifications are shown in Supplementary Table 5. The amplification procedure for PCR was as follows: 94°C for 3 min followed by 30 cycles of 94°C for 30 s, 58°C for 30 s, and 72°C for 1.5 min, followed by a final extension step of 72°C for 20 min.

The resultant DNA fragments and the pBI121 overexpression vector were digested with restriction endonucleases SpeI and XmaI. Then, the PdpapWRKY28 gene was inserted into pBI121 along with the CaMV 35S promoter. The pBI121-PdpapWRKY28 recombinant vector was transferred to Agrobacterium tumefaciens GV3101 by heat shock transformation (Holsters et al., 1978). A. tumefaciens-mediated transformation was used to introduce PdpapWRKY28 into the Pdpap genome (Guo et al., 2018).

### Generating Putative Transformant PdPap

In this study, putative transformant Pdpap lines were obtained using the leaf disc method (Julia et al., 2017). After cutting the youngest leaves of 2-month-old Pdpap plants, leaf discs about 1 cm in diameter were co-cultured with the *Agrobacterium* strain harboring the pBI121-*PdpapWRKY28* recombinant vector. Leaf discs were transferred onto an MS differentiation medium containing 50 mg/L of kanamycin and 200 mg/L of cephalosporin for shoot regeneration. The kanamycin-resistant shoots obtained were rooted on a 0.5X MS medium containing 50 mg/L of kanamycin. After selection by kanamycin, the putative transformants were used for subsequent analysis.

### Molecular Detection of Transformant PdPap

The Super Plant Genomic DNA Kit (Polysacchardes and Polyphenolics-rich) (Tiangen, Beijing, China) was used to extract genomic DNA from the *PdpapWRKY28*-overexpression putative transformant Pdpap lines. Then, PCR detection was performed on the DNA extracted from the putative transformed plants. We used the pBI121-*PdpapWRKY28* plasmid as the positive control and WT Pdpap plants and water as the negative control. For molecular detection of recombinant vectors, we used the primer pairs pBI121-F in the pBI121 vector/*PdpapWRKY28*-R in the gene sequence and *PdpapWRKY28*-F in the gene sequence/pBI121-R in the vector. The primer sequences are shown in Supplementary Table 4. The PCR amplification conditions were as described above.

### Analysis of *PdpapWRKY28* Expression Pattern in Transformants

After 2 months of growth, the transformed *PdpapWRKY28*-overexpression shoots grown in soil were treated with 50 ml *F. oxysporum* (1 × 10^5^/ml) by perfusion into the root. The infection time was 0, 6, 12, 24, or 48 h, and each experimental group had three biological replicates. The entirety of the transformants were frozen in liquid nitrogen and the total RNA of the plants was extracted and reverse transcribed to obtain cDNA. The expression level of the *PdpapWRKY28* gene was determined by qRT-PCR, by, respectively, using WT and transformant cDNA as the template under different pathogen treatment conditions. The primer sequences are shown in Supplementary Table 3. The amplification conditions of qRT-PCR were as described above.

### Growth and Physiological Index Measurements

Two-month-old WT and *PdpapWRKY28*-overexpression putative transformant Pdpap of similar growth statuses were inoculated with *F. oxysporum* for 0, 5, 10, 15, and 20 days as described above. The value of the fresh weight and root length of the WT Pdpap at 0 d was set to 1. The disease status of each plant infected by *F. oxysporum* was observed and the relative fresh weight and root length of the plants were measured. The experiment consisted of three biological replicates.

Two-month-old putative transformants and WT Pdpap of similar growth statuses were inoculated with *F. oxysporum* and sampled at 0, 6, 12, 24, and 48 h. The inoculated plants were immediately frozen with liquid nitrogen after sampling. According to the experimental methods of Cheng (Cheng et al., 2020), the samples were used for the determination of various physiological indicators, including peroxidase (POD) activity, catalase (CAT) activity (Góth, 1991), hydrogen peroxide (H_2_O_2_) content, malondialdehyde (MDA) content, and percentage of electrolyte leakage (Nguyen et al., 2016). The experiment consisted of three biological replicates.

### Antioxidant Capability Test

Nitro blue tetrazolium and 3,3′-diaminobenzidine staining were used to conduct the histochemical detection of H_2_O_2_ and ${\text{O}}_{2}^{-}$ in Pdpap plant tissues (Kumar et al., 2013). Using nitro blue tetrazolium (NBT) can produce insoluble blue products catalyzed by alkaline phosphatase as alkaline phosphatase substrates (Khokon et al., 2011). Darker blue staining indicates that the cells are more damaged by reactive oxygen species (ROS), which means that the antioxidant capacity of the plant cells is relatively lower. On the other hand, 3,3′-diaminobenzidine (DAB) can be dehydrogenated and oxidized to produce a brownish substance through the catalysis of POD (Khokon et al., 2011). In order to assess the damage of F. oxysporum infection to the leaves of Pdpap by ROS, the third to sixth leaves of Pdpap were sampled at 3 days after infection and immediately stained with NBT and DAB according to the experimental methods of Zhou et al. (2020) and Cheng et al. (2020).

### Expression Level of Defense-Associated Genes in PdPap

As described above, PdPap was treated with *F. oxysporum* for 0, 6, 12, 24, and 48 h. We selected two defense-associated genes in salicylic acid (SA) signal transduction pathways and four defense-associated genes in jasmonic acid (JA) signal transduction pathways to perform the qRT-PCR analysis (Huang et al., 2015; Guo et al., 2021). Each experimental group had three biological replicates. The primer sequences are listed in Supplementary Table 6. The amplification conditions of the conducted qRT-PCR were as described above.

### Statistical Analysis

The data were analyzed with the Statistical Software Package for Social Science (SPSS) version 17.0 (Bala, 2016). Using the Student's t-test to compare the data, P < 0.05 was considered significantly different (Choi et al., 2012). In the figures displayed throughout this study, significant differences (P < 0.05) are indicated by different lowercase letters.

## Results

### RNA Sequencing and General Transcription Patterns

Our original data after RNA sequencing contained a few reads with sequencing adapters or low-quality reads. After filtering our sequenced data, 963,059,208 high-quality clean reads were obtained from the 20 samples (Supplementary Table 7). Reads were converted to FPKM. Results of the cluster analysis on duplicated samples in groups and between treatments showed different levels among all samples. The proportion of total reads mapped to the reference genome ranged from 69.28 to 72.55%. Correlation values of duplicated samples within groups were significantly higher than those between treatments (Supplementary Figure 1).

### Differential Expression of PdPap Genes in Response to Infection With *F. oxysporum*

**Figure 1:**
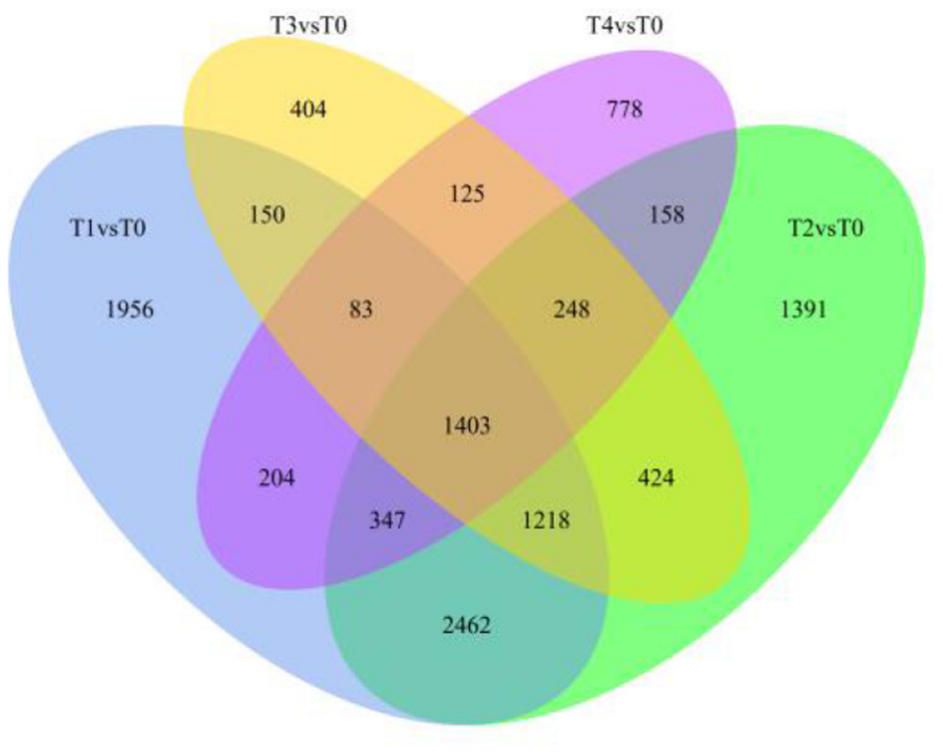
Differential gene Venn map. The sum of the numbers in the Venn diagram represents the total number of differentially expressed genes (DEGs). The overlapping indicates the number of DEGs common in the combinations. T0, T1, T2, T3, and T4 stand for the *Fusarium oxysporum*-treated Pdpap by 0, 6, 12, 24, and 48 h, respectively. The numbers 1, 2, 3, and 4 after the treatment name stands for four biological repetitions of the same treatment operation.

Differentially expressed genes were identified by comparing *F. oxysporum*-treated WT Pdpap with uninfected WT Pdpap at every time (Supplementary Figure 2; Supplementary Table 8). At 6 h after inoculation with *F. oxysporum*, 7,823 differentially expressed genes (DEGs) were associated with the infected plants, namely, 4,431 upregulated and 3,392 downregulated genes. At 12 h after inoculation with *F. oxysporum*, 7,651 DEGs were associated with the infected plants, namely, 4,534 upregulated and 3,177 downregulated genes. At 24 h after inoculation with *F. oxysporum*, 4,055 DEGs were associated with the infected plants, namely, 2,648 upregulated and 1,407 downregulated genes. At 48 h after inoculation with *F. oxysporum*, 3,346 DEGs were associated with the infected plants, namely, 2,292 upregulated and 1,054 downregulated genes. The total number of DEGs between infected and uninfected plants was 11,351 across all tested time points (Figure 1). One thousand four hundred three DEGs were common in the four treatment groups (i.e., hours 6, 12, 24, and 48), accounting for 12.36% of the total DEGs. Of the remaining DEGs, 1,956 were uniquely expressed at 6 h (17.23% of the total DEGs); 1,391 DEGs were uniquely expressed at 12 h (12.25% of the total DEGs); 404 DEGs were uniquely expressed at 24 h (3.56% of the total DEGs); 778 DEGs were uniquely expressed at 48 h (6.85% of total the DEGs). The gene expression patterns of the duplicated samples were similar for each time point (the “groups”) but significantly different between them (the “treatments”) (Supplementary Figure 3).

### Gene Ontology Enrichment Analysis

**Figure 2:**
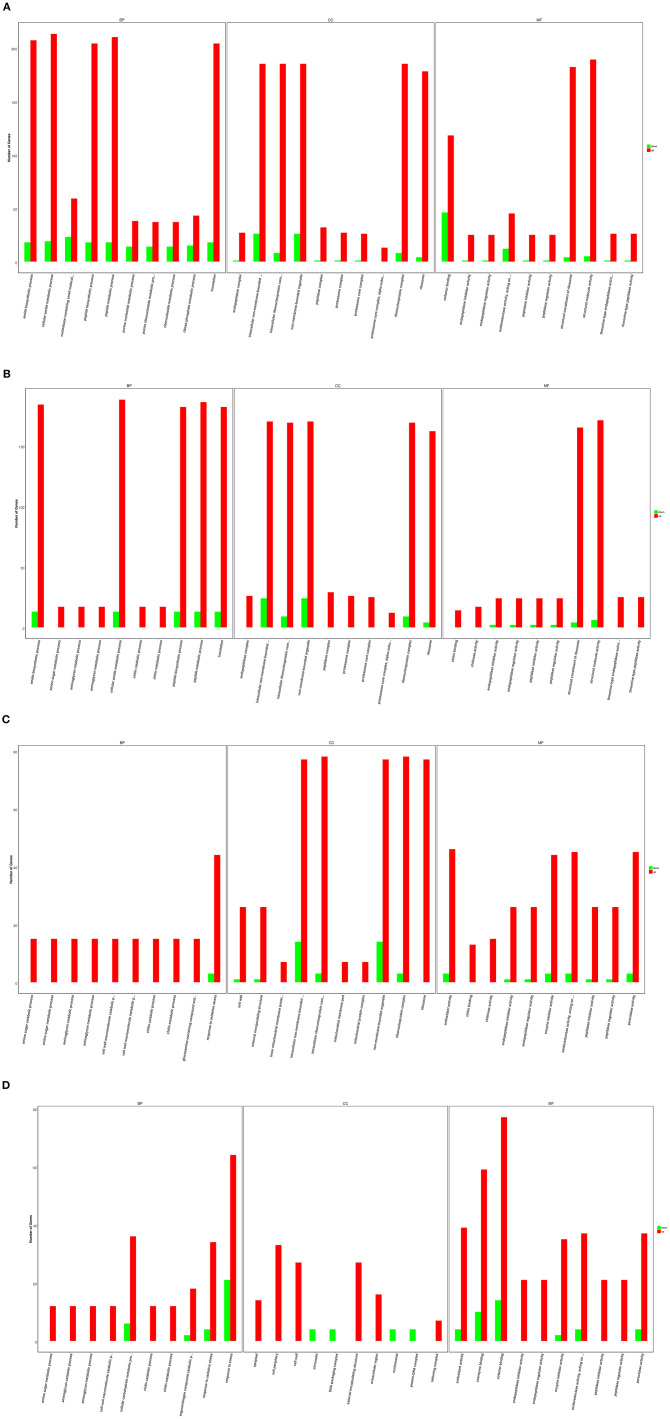
Gene ontology (GO) categories of differentially expressed genes (DEGs) between *Fusarium Oxysporum*-treated and wild type (WT) Pdpap. **(A)** T1 and T0. **(B)** T2 and T0. **(C)** T3 and T0. **(D)** T4 and T0. T0, T1, T2, T3, and T4 stand for the *F. oxysporum*-treated *Populus davidiana* × *P. alba* var. *pyramidalis* Louche (Pdpap) by 0, 6, 12, 24, and 48 h, respectively.

After infection by *F. oxysporum* for 6 h, GO enrichment analysis revealed that the DEGs associated with GO biological processes were mainly distributed within the following GO terms: the cellular amide metabolic process (232 of the DEGs), the peptide metabolic process (228 of the DEGs), the amide biosynthetic process (225 of the DEGs), the translation process (222 of the DEGs), and the peptide biosynthetic process (222 of the DEGs). The DEGs associated with GO cellular components were mainly distributed within the following GO terms: the non-membrane-bounded organelle (211 of the DEGs), the intracellular non-membrane-bounded organelle (211 of the DEGs), the ribonucleoprotein complex (193 of the DEGs), the intracellular ribonucleoprotein complex (193 of the DEGs), and the ribosome (182 of the DEGs). Finally, the DEGs associated with GO molecular functions were distributed within the following GO terms: structural molecule activity (194 of the DEGs), the structural constituent of ribosome (186 of the DEGs), and cofactor binding (164 of the DEGs) (Figure 2A). After infection by *F. oxysporum* for 12 h, the DEGs associated with GO biological processes were mainly distributed within the following GO terms: the cellular amide metabolic process (201 of the DEGs), the peptide metabolic process (199 of the DEGs), the amide biosynthetic process (197 of the DEGs), the translation process (195 of the DEGs), and the peptide biosynthetic process (195 of the DEGs). The DEGs associated with GO cellular components were mainly distributed within the following GO terms: the intracellular non-membrane-bounded organelle (194 of the DEGs), the non-membrane-bounded organelle (194 of the DEGs), the ribonucleoprotein complex (178 of the DEGs), the intracellular ribonucleoprotein complex (178 of the DEGs), and the ribosome (166 of the DEGs). Finally, the DEGs associated with GO molecular functions were distributed within the following GO terms: structural molecule activity (177 of the DEGs) and the structural constituent of ribosome (169 of the DEGs) (Figure 2B). After infection by *F. oxysporum* for 24 h, the DEGs associated with GO biological processes were mainly distributed within the following GO term: the response to oxidative stress process (47 of the DEGs). The DEGs associated with GO cellular components were mainly distributed within the following GO terms: the intracellular non-membrane-bounded organelle (91 of the DEGs), the non-membrane-bounded organelle (91 of the DEGs), the ribonucleoprotein complex (81 of the DEGs), the intracellular ribonucleoprotein complex (81 of the DEGs), and the ribosome (77 of the DEGs). Finally, the DEGs associated with GO molecular functions were distributed within the following GO terms: heme binding (106 of the DEGs) and tetrapyrrole binding (106 of the DEGs) (Figure 2C). After infection by *F. oxysporum* for 48 h, the DEGs associated with GO biological processes were mainly distributed within the following GO terms: response to the stress process (85 of the DEGs), the cellular carbohydrate metabolic process (42 of the DEGs), and response to the oxidative stress process (38 of the DEGs). The DEGs associated with GO cellular components were mainly distributed within the following GO terms: the cell periphery (33 of the DEGs), the cell wall (27 of D the Egs), and the external encapsulating structure (27 of the DEGs). Finally, the DEGs associated with GO molecular functions were distributed within the following GO terms: transferase activity and transferring glycosyl groups (92 of the DEGs), cofactor binding (91 of the DEGs), heme binding (83 of DEGs), and tetrapyrrole binding (83 of the DEGs) (Figure 2D).

Results showed that infection by *F. oxysporum* affected the expression of many genes related to stress response (Supplementary Table 9), including four universal stress protein (USP) A-like proteins (LOC18109283, LOC7496605, LOC7464025, and LOC7494517), 9 P450 proteins (LOC7464737, LOC7480159, LOC7463864, LOC7475048, LOC7454262, LOC112326669, LOC18099663, LOC18099865, and LOC18104817), and 10 WRKY proteins (Supplementary Table 10).

### Validation of DEGs by qRT-PCR

**Figure 3:**
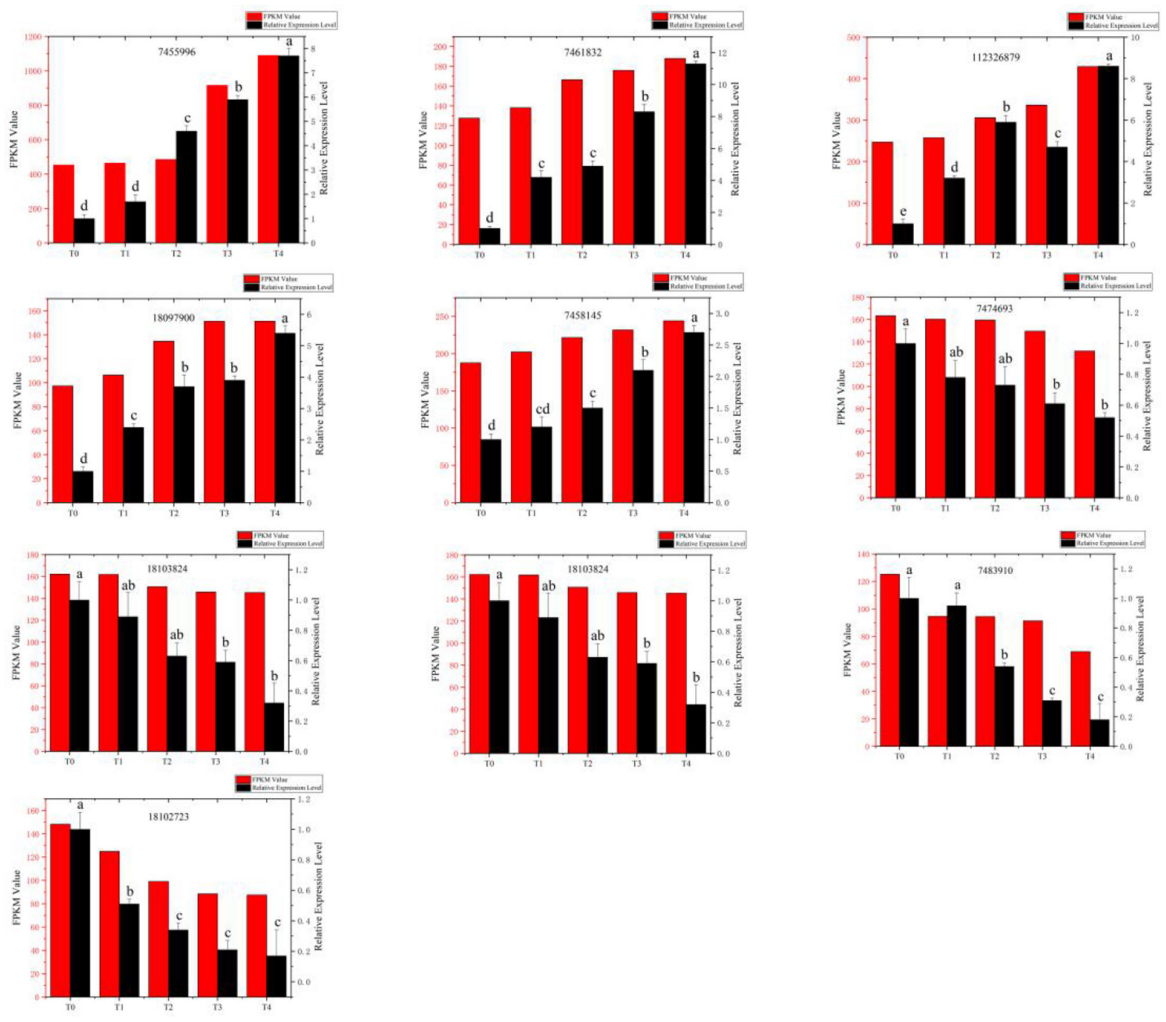
Comparison of relative expression of differential genes detected by RNA-Seq and quantitative real-time polymerase chain reaction (qRT-PCR). Red bars: The change in the fragments per kilobase million (FPKM) value obtained by RNA sequencing (left vertical axis). Black bars: The relative expression level (right vertical axis) obtained by qRT-PCR experiment. T0, T1, T2, T3, and T4 stand for the *Fusarium oxysporum*-treated Pdpap by 0, 6, 12, 24, and 48 h, respectively.

Five upregulated and five downregulated DEGs were randomly selected for qRT-PCR verification. Results showed that upregulated and downregulated expression patterns of genes were consistent with the patterns obtained by RNA sequencing. Therefore, these qRT-PCR results support the gene expression levels detected during RNA sequencing (Figure 3).

### Screening of Genes Related to Disease Resistance and Expression Pattern Analysis

As previously explained, DEGs were screened according to log_2_ (FoldChange) > 3 and *P* adj < 0.05. The genes with increased expression levels during infection by *F. oxysporum* were obtained (Supplementary Table 11). It can be seen that 36 genes in Pdpap showed a continuously upregulated expression trend upon infection with *F. oxysporum*. Among these upregulated genes, various transcription factors were obtained, such as ethylene-responsive factors (ERFs), WRKYs, and V-MYB (avian myeloblastosis virus oncogenes).

In order to clarify the role of the transcription factors *PdpapWRKY28, PdpapERF6*, and *PdpapMYB41* in response to *F. oxysporum* infection of Pdpap, we analyzed expression levels of these three genes in Pdpap treated with *F. oxysporum* (Supplementary Figure 4). After Pdpap was infected by *F. oxysporum*, the expression of the *PdpapWRKY28* gene continued to significantly increase. Its expression level reached its maximum 48 h after infection. Contrarily, the expression levels of *PdpapERF6* and *PdpapMYB41* both decreased during different periods. Based on the expression pattern of the *PdpapWRKY28* gene after infection with *F. oxysporum*, combined with the research of Wu et al. (2011) and Chen et al. (2013) on the function of the *WRKY28* gene, we speculated that overexpression of the *PdpapWRKY28* gene may play an important role in the response of Pdpap to *F. oxysporum*.

### Phylogenetic Tree Analysis

**Figure 4:**
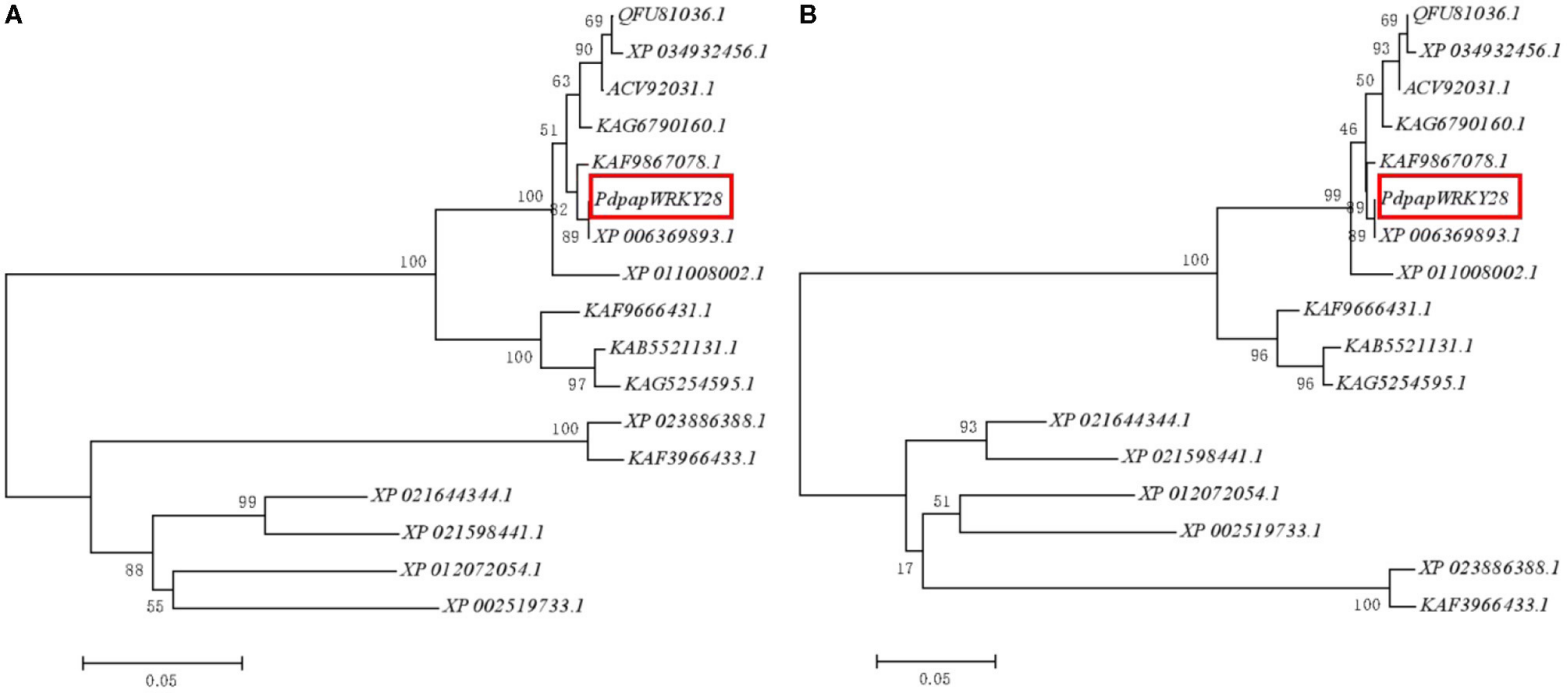
Dendrogram of the *PdPapWRKY28* gene family proteins. **(A)** Neighbor joining (NJ)-phylogenetic tree; **(B)** Maximum likelihood (ML)-phylogenetic tree. The dendrogram was constructed using the MEGA5 with the neighbor method and Poisson model. The *PdPapWRKY28* gene is marked with a red frame, and the results showed the same evolutionary relationship under the two algorithms.

A 977 bp cDNA fragment of *PdpapWRKY28* was cloned from WT Pdpap. Sixteen (16) WRKY protein sequences similar to the *PdpapWRKY28* gene were downloaded (see the Methods section for more details) and compared. Through the construction of a neighbor joining (NJ) phylogenetic tree, the putative evolutionary relationships among the WRKY sequences were initially revealed. Poisson bootstrap resampling was used to bolster the reliability of the NJ results, the results of which are shown in Figure 4A. In order to further confirm the reliability of the NJ results, we built a maximum likelihood (ML) phylogenetic tree using the same sequence data. The ML phylogenetic tree is shown in Figure 4B. By visually comparing the results of the two phylogenetic trees, the two models have good consistency. Additionally, the *PdpapWRKY28* gene shares 100% sequence identity with the *WRKY28* gene from *P. trichocarpa* (XP_006369893.1). The *PdPapWRKY28* gene also shares high sequence homology with similar proteins from other species, such as *Populus trichocarpa* (100%, XP_006369893.1), *P. deltoides* (99.04%, KAF9867078.1), *P. tomentosa* (98.4%, KAG6790160.1), (*P. tomentosa* x *P. bolleana*) x *P. tomentosa* (98.08%, ACV92031.1), *P. alba* var. *pyramidalis* (97.76%, QFU81036.1), *P. alba* (97.44%, XP_034932456.1), *P. euphratica* (97.44%, XP_011008002.1), *Salix dunnii* (87.58%, KAF9666431.1), *S. brachista* (86.03%, KAB5521131.1), *S. suchowensis* (85.71%, KAG5254595.1), *Hevea brasiliensis* (71.21%, XP_021644344.1), *Jatropha curcas* (68.81%, XP_012072054.1), *Ricinus communis* (68.75%, XP_002519733.1), *Manihot esculenta* (66.67%, XP_021598441.1), *Quercus suber* (66.25%, XP_023886388.1), *Castanea mollissima* (65.94%, KAF3966433.1).

### Tissue-Specific Differential Expression of the *PdpapWRKY28* Gene

Quantitative RT-PCR was used to analyze the spatial expression profiles of the *PdpapWRKY28* gene in the different tissues of the Pdpap plants. From the results of the analysis, it can be seen that the expression level of the *PdpapWRKY28* gene is lowest in leaves and highest in stems (Supplementary Figure 5A). We also compared expression levels in different parts of the stems, with the results showing that the expression of the *PdpapWRKY28* gene was lowest in the midsection of the stems and highest at the base of the stems (Supplementary Figure 5B).

### Molecular Detection of Overexpressing Putative Transformants of *PdpapWRKY28*

After the overexpression vector of *PdpapWRKY28* was transformed into WT Pdpap, a total of five putative transformant lines were obtained. Then, DNA was extracted from the WT and putative transformants and PCR was performed with the primer pairs pBI121-F/*PdpapWRKY28*-R and *PdpapWRKY28*-F/pBI121-R. After PCR identification, each line containing DNA fragments was amplified by the two pairs of primers. The detection result is shown in Supplementary Figure 6. It can be seen from the figure that the bands in lanes 1-1 to 5-2 are the same as the bands in lanes “+-1” and “+-2,” and that a single amplified fragment was detected in each lane. There are no amplified bands in lanes “−1,” “−2,” and “w-1” and “w-2.” Gel electrophoresis showed that the sizes of the amplified target bands obtained from the five putative transformant lines were as expected. The recombinant plasmid (positive control) indicated that the gene was only amplified in the transformants but not in the WT Pdpap. This indicates that the pBI121-*PdpapWRKY28* vector was successfully transformed into the WT Pdpap. The five transformants detected were found to contain the *PdpapWRKY28* gene under the control of the CaMV 35S promoter.

### Expression Level Analysis of *PdpapWRKY28* in Transformants

**Figure 5:**
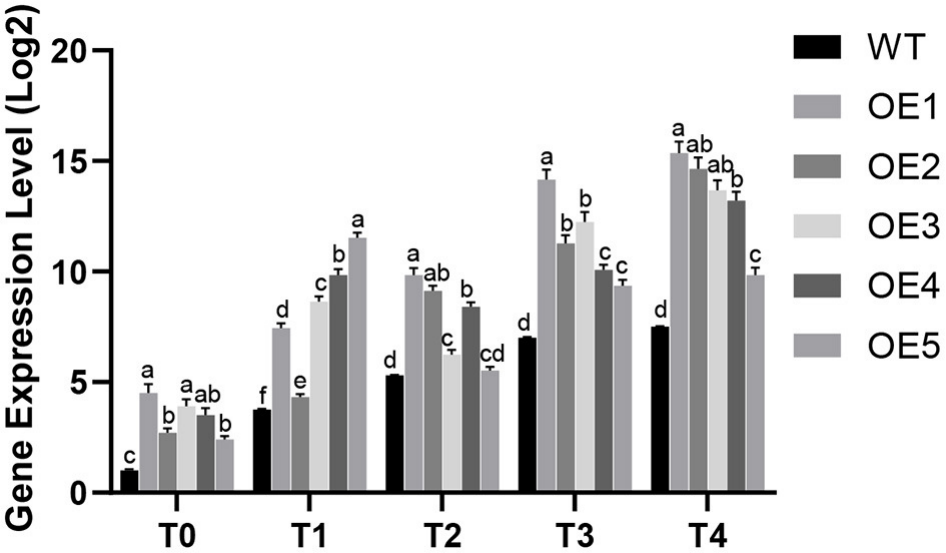
The expression level analysis of *PdPapWRKY28* in putative transformants. WT: wild type *Populus davidiana* × *P. alba* var. *pyramidalis* Louche (Pdpap). OE1–OE5: transformant lines. T0–T4: infection time was 0, 6, 12, 24, and 48 h. Error bars represented the standard deviation of the three independent replicates. Significant differences (*P* < 0.05) were indicated by different lowercase letters.

Quantitative RT-PCR analysis was performed on five putative *PdpapWRKY28*-overexpression transformants under different infection conditions. The experimental result is shown in Figure 5. It can be seen that the expression level of the *PdpapWRKY28* gene in the putative overexpressing transformants under different infection conditions was about 1.04–4.5 times higher than that in the WT Pdpap. Of these five putative transformants, the overexpression lines OE1 and OE3, with the highest and middle expression levels, respectively, were selected as plant materials for subsequent functional verification.

### Resistance Analysis of Transformants to *F. oxysporum* Infection

**Figure 6:**
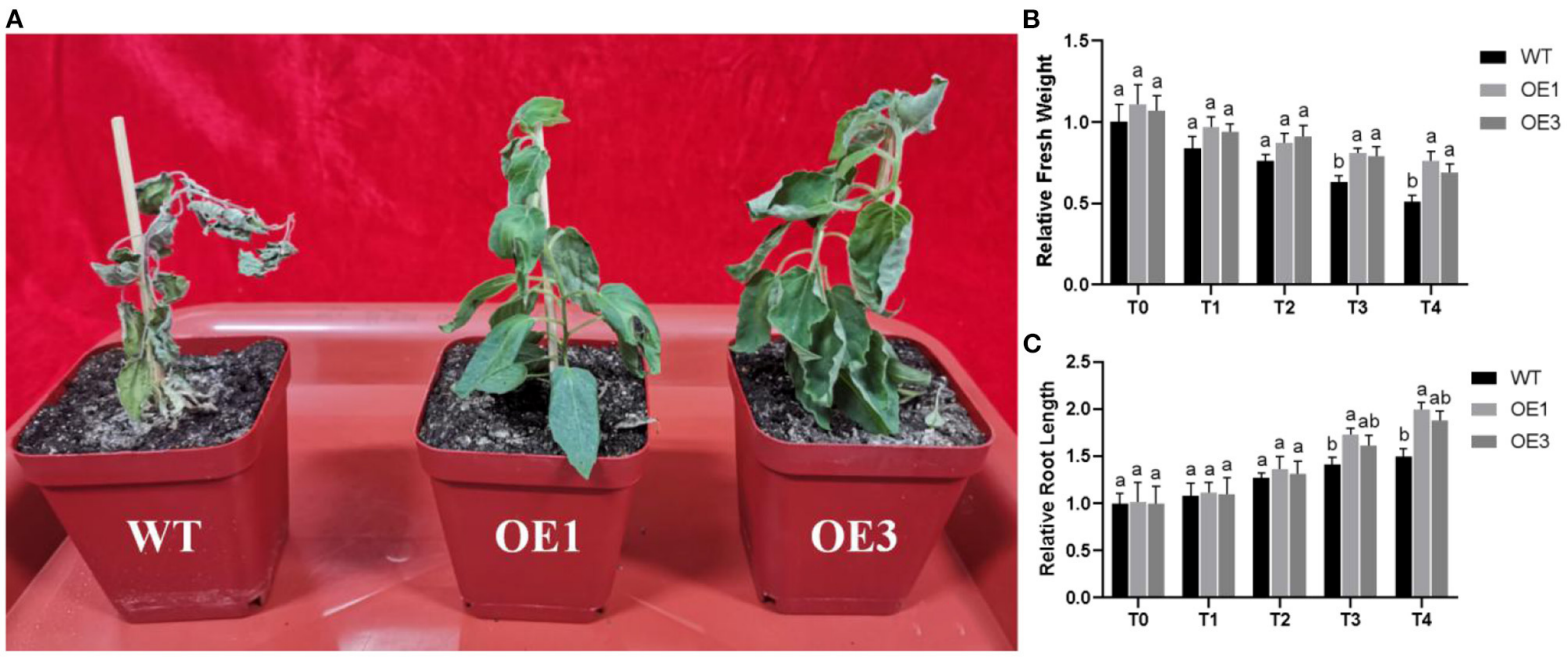
**(A)** The growth status of WT and *PdPapWRKY*-overexpression putative transformants OE1 and OE3 lines inoculated with *Fusarium oxysporum*; **(B)** Relative fresh weights of WT and transformants treated with *F. oxysporum*; **(C)** Root lengths of WT and transformants treated with *F. oxysporum*. WT: wild type Pdpap; OE1, OE3: transformant lines. T0–T4: infection time was 0, 5, 10, 15, and 20 days. The error bars represented the standard deviation of the three independent replicates. Significant differences (*P* < 0.05) were indicated by different lowercase letters.

To test resistance to *F. oxysporum* under natural conditions, WT and *PdpapWRKY28-*overexpression transformants OE1 and OE3 were all inoculated with *F. oxysporum* and grown in an artificial climate room for 10 d. In Figure 6A, it can be seen that after inoculation, WT Pdpap wilted and died, but the *PdpapWRKY28*-overexpression transformants (OE1 and OE3) grew well, although there was evidence of slight water loss in their leaf blades and petioles. This experimental result shows that the overexpression of *PdpapWRKY28* in Pdpap yields stronger resistance against *F. oxysporum* infection compared with WT Pdpap.

The relative fresh weights and root lengths of WT and transformants inoculated with *F. oxysporum* at 0, 5, 10, 15, and 20 days were determined, and the weights of WT Pdpap inoculated with *F. oxysporum* at 0 day were set to 1. The relative fresh weights and root lengths are shown in Figures 6B,C. The relative fresh weights and root lengths of the two transformants were basically consistent with WT Pdpap at T0. In contrast, after being inoculated with *F. oxysporum*, the transformants were superior to the WT Pdpap under the same infection conditions, indicating that transformants grow better after infection with *F. oxysporum*. The results show that overexpressed expression of the *PdpapWRKY28* gene can enhance resistance to *F. oxysporum* in the early stages of Pdpap growth.

### Physiological Analysis of Transformants Infected With *F. oxysporum*

**Figure 7:**
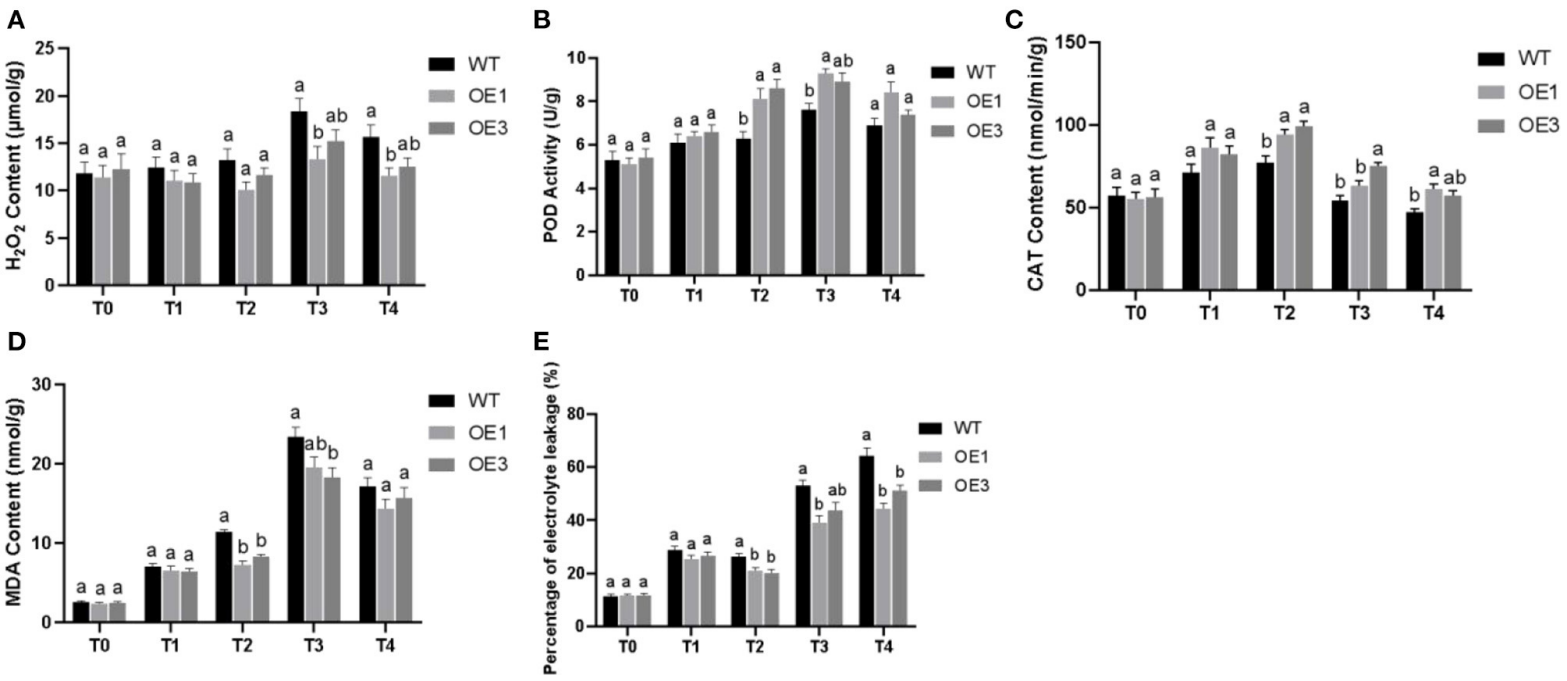
The physiological characteristic analysis of wild type (WT) and *PdPapWRKY28*-overexpression putative transformants treated with *Fusarium oxysporum*. Two transformants and WT were used as biological replicates for physiological analysis. **(A)** The result of hydrogen peroxide (H_2_O_2_) content measurements; **(B)** The result of peroxidase (POD) activity measurements; **(C)** The result of catalase (CAT) activity measurements; **(D)** The result of malondialdehyde (MDA) contents; **(E)** The result of electrolyte leakage percentage. T0–T4: infection time was 0, 6, 12, 24, and 48 h. Error bars represented the standard deviation of the three independent replicates. Significant differences (*P* < 0.05) were indicated by different lowercase letters.

The results of our H_2_O_2_ measurements are shown in Figure 7A. The experiment included three biological replicates. Prior to infection, the H_2_O_2_ content in WT Pdpap was 1 ± 0.04-fold higher than that in the transformants. Under infection with *F. oxysporum*, the relative differences were 1.13 ± 0.01-fold, 1.22 ± 0.09-fold, 1.30 ± 0.09-fold, and 1.31 ± 0.05-fold at 6, 12, 24, and 48 h, respectively. At T0, the H_2_O_2_ content of the two transformants (OE1 and OE3) were similar to that in the WT Pdpap. However, after infection with *F. oxysporum*, the H_2_O_2_ content in *PdpapWRKY28-*overexpression transformants was lower than that in the Paparchy WT Pdpap under the same treatment conditions. This result indicates that the OE1 and OE3 lines had stronger resistance to *F. oxysporum* than the WT Pdpap did.

The results of our POD activity measurements are shown in Figure 7B. The experiment included three biological replicates. Prior to infection, the POD activity of the transformants was 0.99 ± 0.03-fold higher than that of the WT Pdpap. The relative differences were 1.07 ± 0.02-fold, 133 ± 0.04-fold, 1.20 ± 0.03-fold, and 1.15 ± 0.08-fold at 6, 12, 24, and 48 h, respectively. At T0, the POD activity in the transformants is similar to that in the WT Pdpap. After infection with *F. oxysporum*, the POD activity in transformants was higher than that in the WT Pdpap under the same treatment conditions. This result indicates that *PdpapWRKY28*-overexpression transformants had greater resistance to the pathogen than the WT Pdpap.

The results of our CAT activity measurements are shown in Figure 7C. The experiment had three biological replicates. Prior to infection, the CAT activity in the transformants was 0.97 ± 0.01-fold higher than that in the WT Pdpap. After infection by *F. oxysporum*, the relative differences were 1.18 ± 0.03-fold, 1.26 ± 0.04-fold, 1.28 ± 0.11-fold, and 1.26 ± 0.05-fold at 6, 12, 24, and 48 h, respectively. It can be seen that, at T0, the CAT activity in the two transformants is similar to that in the WT Pdpap. However, after infection with *F. oxysporum*, the CAT activity of the transformants was higher than that of the WT Pdpap in the same infection conditions. This result provides evidence that *PdpapWRKY28*-overexpression transformants have higher resistance to *F. oxysporum* than WT Pdpap.

The results of our MDA content measurements are shown in Figure 7D. The experiment included three biological replicates. Prior to infection, the MDA content of the WT Pdpap was 1.06 ± 0.02-fold higher than that in the transformants. Additionally, WT Pdpap was 1.1 ± 0.02-fold, 1.47 ± 0.1-fold, 1.24 ± 0.05-fold, and 1.15 ± 0.05-fold higher than that in the transformants at 6, 12, 24, and 48 h, respectively, after infection by *F. oxysporum*. It can be seen that, at T0, the MDA content of the transformants was similar to that of WT Pdpap. After infection with *F. oxysporum*, however, the transformants had consistently lower malondialdehyde content than that of the WT Pdpap subjected to the same infection inoculation dosage and duration. This result indicates that *PdpapWRKY28*-overexpression transformants had increased resistance to *F. oxysporum* compared to WT Pdpap.

The percentage of electrolyte leakage is shown in Figure 7E. Prior to *F. oxysporum* inoculation, electrolyte leakage in the WT Pdpap was 0.97 ± 0.01-fold higher than that in the transformants. After infection with *F. oxysporum*, the percentage of electrolyte leakage in the transformants was 1.1 ± 0.03-fold, 1.28 ± 0.03-fold, 1.29 ± 0.07-fold, and 1.35 ± 0.1-fold lower than in the WT Pdpap at 6, 12, 24, and 48 h, respectively. It can be seen that, at T0, the percentage of electrolyte leakage in the two transformants was similar to that in the WT Pdpap. After infection with *F. oxysporum*, however, the percentage of electrolyte leakage in the transformants was lower than that in the WT Pdpap subjected to the same inoculation dosage and duration. This result indicates that *PdpapWRKY28*-overexpression transformants had stronger resistance to *F. oxysporum* infection than the WT Pdpap.

### Histochemical Staining

**Figure 8:**
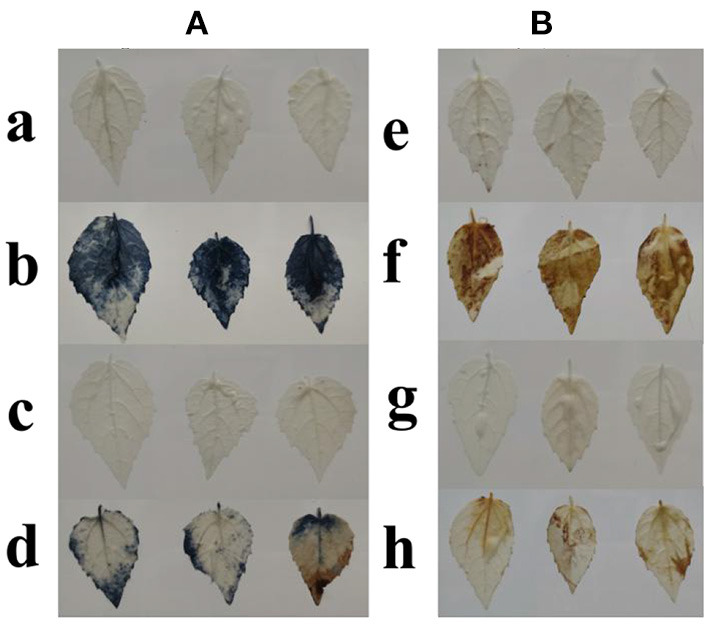
Histochemical staining by nitro blue tetrazolium (NBT) and 3,3′-diaminobenzidine (DAB). **(A)** NBT staining. a: Leaves of wild type (WT) *Populus davidiana* × *P. alba* var. *pyramidalis* Louche (PdPap) without infection, b: Leaves of WT PdPap with *Fusarium oxysporum*, c: Leaves of transformants without *F. oxysporum*, d: Leaves of transformants with *F. oxysporum;* **(B)** DAB staining. e: Leaves of WT PdPap without infection, f: Leaves of WT PdPap with *F. oxysporum*, g: Leaves of transformants without *F. oxysporum*, h: Leaves of transformants with *F. oxysporum*. Staining intensities of WT and transformants under infection of *F. oxysporum* are similar, but greatly increased in WT infected by *F. oxysporum*.

The accumulation of superoxide anions (${\text{O}}_{2}^{-}$) and H_2_O_2_ in WT and transformants was analyzed by means of NBT and DAB staining of Pdpap leaves infected with *F. oxysporum* (Figure 8). From the degree of NBT staining, it could be seen that the leaves of infected *PdpapWRKY28*-overexpression transformants (Figures 8A-d) were slightly darker than those of uninfected WT Pdpap (Figures 8A-a) and transformants (Figures 8A-c), but the overall appearance was similar. However, the difference in color compared with leaves of WT Pdpap infected by *F. oxysporum* was striking (Figures 8A-b). Likewise, the degree of DAB staining of the leaves of infected *PdpapWRKY28*-overexpression transformants (Figures 8B-h) was slightly darker than that of uninfected WT Pdpap plants (Figures 8B-e) and transformants (Figures 8B-g), but the overall appearance was similar. However, the difference in color compared with leaves of WT Pdpap infected by *F. oxysporum* is obvious (Figures 8B-f). These results indicate that the tissues in transformants showed much less damage than those in WT Pdpap after both were infected with *F. oxysporum*. It can be seen that leaf cells in transformants have a stronger ability to remove ROS, including O^2−^ and H_2_O_2_, thereby reducing cell damage and enhancing plant tolerance. This result is consistent with the POD and CAT activity measurements, which provides further evidence that the overexpression of the *PdpapWRKY28* gene in Pdpap likely reduces the accumulation of intracellular ROS. Thus, *PdpapWRKY28*-overexpression transformants could very effectively enhance the antioxidant capacity of Pdpap plants and improve their resistance to *F. oxysporum*.

### Differential Expression Analysis of Defense-Associated Genes in PdPap

**Figure 9:**
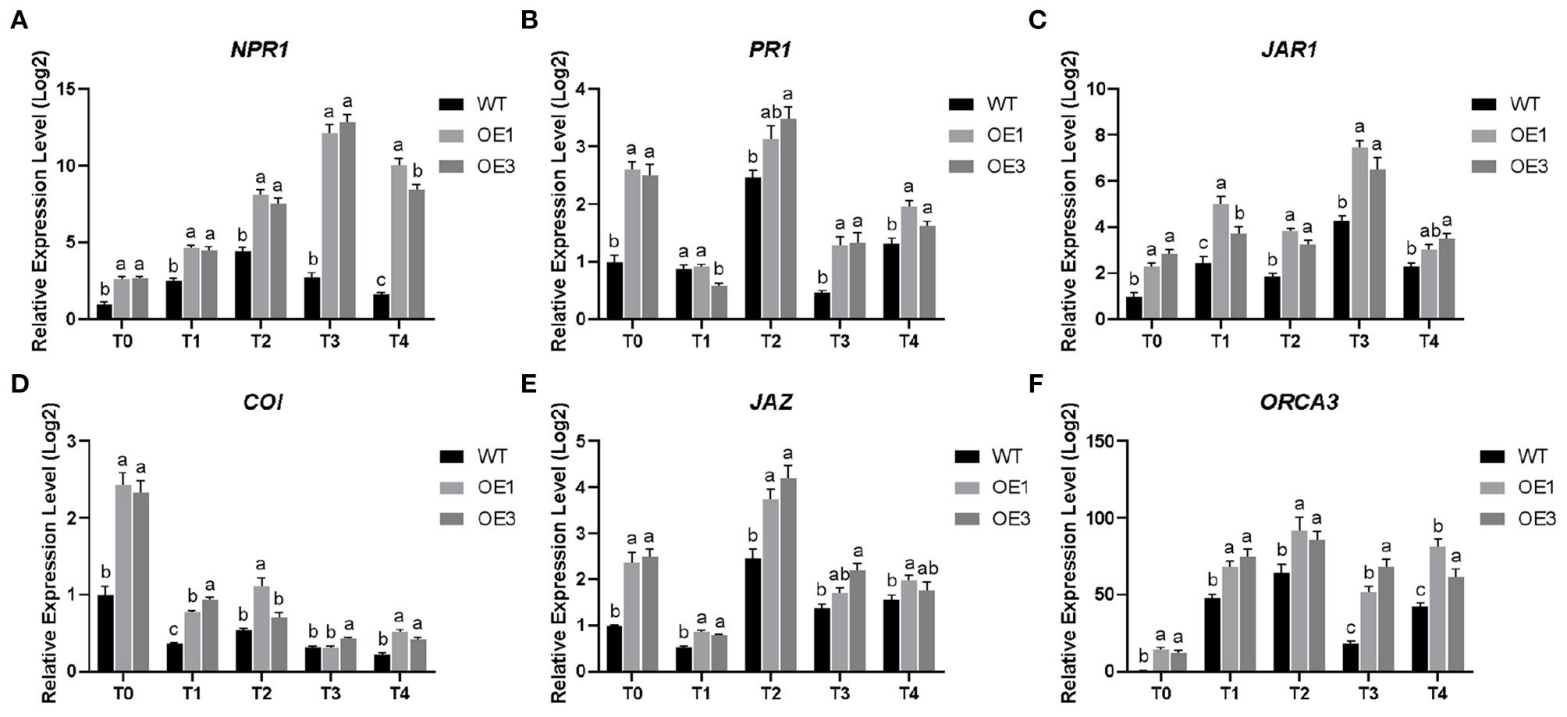
Differential expression level of six defense-associated genes in *Populus davidiana* × *P. alba* var. *pyramidalis* Louche (PdPap) after infection with *Fusarium oxysporum*. **(A)** nonexpressor of pathogenesis-related genes (*NPR1*); **(B)** pathogenesis-related protein genes (*PR1*); **(C)** jasmonic acid resistant gene (*JAR1*); **(D)** coronatine insensitive protein gene (*COI*); **(E)** jasmonate ZIM-domain gene (*JAZ*); **(F)** Octadecaniod-derivative response AP2 domain (*ORCA3*). T0–T4: Infection time was 0, 6, 12, 24, and 48 h. Error bars represented the standard deviation of the three independent replicates. Significant differences (*P* < 0.05) were indicated by different lowercase letters. WT: wildtype Pdpap; OE1, OE3: transformant lines.

To further understand the regulatory role of the *PdpapWRKY28* gene in response to *F. oxysporum* infection, we used qRT-PCR to detect expression levels of hormone signal pathway genes related to plant defense (Figure 9).

The SA signal transduction pathway mainly includes the non-expressor of pathogenesis-related genes (*NPR1*) and pathogenesis-related protein genes (*PR1*). In this study, the expression level of *NPR1* gene showed a trend of upregulation first and then downregulation (Figure 9A). The expression level of the *NPR1* gene in the WT Pdpap reached its highest at 12 h and was 4.46-fold higher than that in control. The expression levels of the *NPR1* gene in the OE1 and OE3 lines reached their highest at 24 h. They were 12.14-fold and 12.87-fold higher than that in control, respectively. The expression level of the *PR1* gene was upregulated during 6–12 h and the later stage of infection and reached its highest at 12 h (Figure 9B). The expression level of the *PR1* gene in the WT Pdpap was 2.46-fold higher than that in control. The expression levels of the *PR1* gene in the OE1 and OE3 lines were 3.13-fold and 3.48-fold higher than that in control, respectively. Therefore, *PdpapWRKY28* gene could induce the SA signal transduction pathway in Pdpap.

The JA signal transduction pathway mainly includes the jasmonic acid resistant gene (*JAR1*), the coronatine insensitive protein gene (*COI*), the jasmonate ZIM-domain gene (*JAZ*), and the Octadecaniod-derivative response AP2 domain gene (*ORCA3*). After infection by *F. oxysporum*, the expression level of the *JAR1* gene showed an overall trend of upregulation first and then downregulation. It reached highest at 24 h (Figure 9C). The expression level of the *JAR1* gene in the WT Pdpap was 4.29-fold higher than that in control. The expression levels of the *JAR1* gene in the OE1 and OE3 lines were 7.46-fold and 6.5-fold higher than that in control, respectively. The expression level of the *COI* gene was downregulated and reached its lowest at 48 h (Figure 9D). The expression level of the *COI* gene in the WT Pdpap was 0.231-fold lower than that in control. The expression levels of the *COI* gene in the OE1 and OE3 lines were 0.519-fold and 0.42-fold lower than that in control, respectively. The expression level of the *JAZ* gene showed an overall trend of upregulation first and then down-egulation. It reached its highest at 12 h (Figure 9E). The expression level of the *JAZ* gene in the WT Pdpap was 2.46-fold higher than that in control. The expression levels of the *JAZ* gene in the OE1 and OE3 lines were 3.73-fold and 4.2-fold higher than that in control, respectively. The expression level of the *ORCA3* gene was upregulated first and then downregulated. It reached its highest at 12 h (Figure 9F). The expression level of the *ORCA3* gene in the WT Pdpap was 64.45-fold higher than that in control. The expression levels of the *ORCA3* gene in the OE1 and OE3 lines were 92.04-fold and 85.79-fold higher than that in control, respectively. Therefore, the *PdpapWRKY28* gene could induce the JA signal transduction pathway in Pdpap.

## Discussion

It is well-supported that the response of plants to adversity requires the joint action of multiple genes (Li et al., 2015). In that regard, the results of this are consistent with previous studies. These previous studies used RNA sequencing methods to understand the response patterns of plants affected by pathogens (Rosli et al., 2013; Zeng et al., 2014; Sucher et al., 2017). However, little is known about the processes and mechanisms of Pdpap responses to *F. oxysporum*.

Analysis and identification of the genes involved in responses to *F. oxysporum* are essential for understanding the mechanisms of root rot and adopting effective control strategies for the disease. Under *F. oxysporum* treatment, changes in transcript levels in Pdpap directly reflect the response of Pdpap to *F. oxysporum*.

Transcription factors are important for abiotic and biological stress responses (Akhtar et al., 2012). It has been found that transcription factor families such as WRKY, AP2/ERF, NAC, bZIP, and MYB (Pu et al., 2019) affect stress tolerance in plants by regulating downstream stress response genes (Joshi et al., 2016). In particular, WRKY transcription factors are involved in diverse secondary metabolism and plant growth pathways, such as trichome development, tannin production in the seed coat, seed coat development, root development, embryogenesis, and leaf senescence (Johnson et al., 2002; Lagace and Matton, 2004). These WRKYs are also involved in the response of plants to a variety of environmental stresses, such as pathogens, low temperature, salt, and drought (Huang and Duman, 2002; Yoda et al., 2002).

There are several reports on the resistance functions of the *PdpapWRKY28* gene in response to pathogens (Ülker and Somssich, 2004; Wu et al., 2011), salt stress (Babitha et al., 2013; Wang et al., 2020), and acids (Chen et al., 2013). These studies established a theoretical foundation for our early prediction and subsequent validation experiments. Studies showed that *AtWRKY28* participates in the salicylic acid (SA)-mediated signal pathway of disease resistance and plays an important role in SA metabolism by regulating the expression of isochorismate synthase (ISCI) genes (Verk et al., 2011). Therefore, we hypothesized that the *PdpapWRKY28* gene might participate in the regulation of the SA signaling pathway to promote the effective response of Pdpap to *F. oxysporum* infection.

We observed differential expression levels of *PdpapWRKY28* in the transformants. This may have been due to different copy numbers and/or insertion sites of the gene as mediated by the recombinant vector pBI121-*PdpapWRKY28*. We will analyze the copy numbers and insertion sites of *PdpapWRKY28* in putative transformants in a follow-up study. By observing the responses of the *PdpapWRKY28*-overexpression transformants OE1 and OE3, we thought that the overexpression of the *PdpapWRKY28* played a significant role in the growth and health status of the plants after pathogen infection. The overexpression of *PdpapWRKY28* could effectively reduce the accumulation of ROS in Pdpap and promote the activities of defense-related enzymes to protect plants from external stress. In the future, we will further study the molecular mechanism of gene regulation and the gene network related to *PdpapWRKY28* expression in response to infection by *F. oxysporum*.

To determine the molecular mechanism of Pdpap in response to *F. oxysporum* after *PdpapWRKY28-*overexpression, we analyzed the expression level of hormone signal transduction genes related to plant disease resistance. The SA signal transduction pathway mainly includes *NPR1* and *PR1* (Shah et al., 1999). The *NPR1* gene regulates the transcription of *PR1* gene. High transcription level of the *PR1* gene can eventually stimulate plants to establish systemic acquired resistance (SAR) (Ali et al., 2017). Furthermore, JA is an important signal molecule of induced systemic resistance (ISR). The JA signal transduction pathway mainly includes *JAR1, COI, JAZ*, and *ORCA3* (Gfeller et al., 2010). By regulating the transcription of *JAR1, COI*, and *JAZ*, the expression of *ORCA3* can be regulated (Choudhary et al., 2007). Finally, it stimulates the expression of plant defense proteins and synthesis of secondary metabolites, which could stimulate plants to establish ISR (Choudhary et al., 2007). In this study, the expression level of *NPR1* and *PR1* was negatively correlated during 0–48 h. The regulation of *NPR1* inhibited the expression of *PR1*.Therefore, the overexpression of the *PdpapWRKY28* gene did not ultimately stimulate the SA signal in Pdpap. Furthermore, Pdpap did not establish SAR. The expression level of *COI* was negatively correlated with *JAR1* and *ORCA3* during 0–48 h. Under the induction of *PdpapWRKY28-*overexpression, the *JAR1* gene was positively regulated. The *COI* gene was negatively regulated. The expression level of *JAZ* rose first and then declined. They eventually activated the expression of *ORCA3*. Therefore, the overexpression of *PdpapWRKY28* gene can stimulate the JA signal of Pdpap, thereby establishing ISR in Pdpap.

The focus of this paper was to explore molecular response patterns of Pdpap treated with *F. oxysporum*. *PdpapWRKY28* was identified and its response patterns were verified. The study also provides a theoretical basis for enhancing resistance of Pdpap to *F. oxysporum*. These findings will provide a critical gene for reference in research on root rot resistance breeding in the future.

## Data Availability Statement

The original contributions presented in the study are publicly available. This data can be found here: National Center for Biotechnology Information (NCBI) BioProject database under accession number PRJNA741264.

## Author Contributions

JD: investigation, methodology, and writing of the draft. JW: formal analysis, funding acquisition, investigation, and project administration. PZ: formal analysis and validation. XH: formal analysis and methodology. YW: formal analysis. LL: resources. YZ: supervision. WM: writing, including review and editing. LM: funding acquisition, methodology, and project administration. All authors contributed to the article and approved the submitted version.

## Conflict of Interest

The authors declare that the research was conducted in the absence of any commercial or financial relationships that could be construed as a potential conflict of interest.

## Publisher's Note

All claims expressed in this article are solely those of the authors and do not necessarily represent those of their affiliated organizations, or those of the publisher, the editors and the reviewers. Any product that may be evaluated in this article, or claim that may be made by its manufacturer, is not guaranteed or endorsed by the publisher.

## Supplementary Material

Supplementary Figure 1 General transcription patterns of all samples. Pearson correlation between *Fusarium oxysporum*-treated and wild type (WT) *Populus davidiana* × *P. alba* var. *pyramidalis* Louche (Pdpap). T0, T1, T2, T3, and T4 stand for the *F. oxysporum*-treated Pdpap by 0, 6, 12, 24, and 48 h, respectively. The numbers 1, 2, 3, and 4 after the treatment name stand for the four biological repetitions of the same treatment operation.

Supplementary Figure 2 Differential gene volcano map. T0, T1, T2, T3, and T4 stands for the *F. oxysporum*-treated *Populus davidiana* × *P. alba* var. *pyramidalis* Louche (Pdpap) by 0, 6, 12, 24, and 48 h, respectively. The numbers 1, 2, 3, and 4 after the treatment name stand for the four biological repetitions of the same treatment operation.

Supplementary Figure 3 Heat map of all the differentially expressed genes (DEGs) between the *Fusarium oxysporum*-treated and wild type (WT) groups of *Populus davidiana* × *P. alba* var. *pyramidalis* Louche (Pdpap). T0, T1, T2, T3, and T4 stand for the *F. oxysporum*-treated Pdpap by 0, 6, 12, 24, and 48 h, respectively. The numbers 1, 2, 3, and 4 after the treatment name stand for the 4 biological repetitions of the same treatment operation.

Supplementary Figure 4 Expression patterns analysis of candidate genes. The relative expression levels are obtained from the quantitative real-time PCR (qRT-PCR) measurements of *Populus davidiana* × *P. alba* var. *pyramidalis* Louche (Pdpap). Significant differences (*P* < 0.05) were indicated by different lowercase letters. Error bars represented the standard deviation of the three independent replicates.

Supplementary Figure 5 Tissue-specific differential expression of the *PdPapWRKY28* gene. **(A)** In different tissues of *Populus davidiana* × *P. alba* var. *pyramidalis* Louche (Pdpap); **(B)** In different parts of stems. The relative expression levels were obtained from the quantitative real-time PCR (qRT-PCR) measurements of Pdpap. Significant differences (*P* < 0.05) were indicated by different lowercase letters. Error bars represented the standard deviation of the three independent replicates.

Supplementary Figure 6 Molecular detection in the putative transformants *Populus davidiana* × *P. alba* var. *pyramidalis* Louche (Pdpap) of *PdPapWRKY28*. M: DL2,000 Marker; 1–5: Five overexpressing putative transformant lines were used as amplification templates; +: Positive control using pBI121-*PdPapWRKY28* plasmid as template; -, Negative control with water as template; W, Negative control with WT Pdpap as template;−1, PCR detection of putative transformant lines using pBI121-F and *PdPapWRKY28*-R as primers;−2, PCR detection of putative transformant lines with *PdPapWRKY28*-F and pBI121-R as primers.

Supplementary Table 1 Primer sequences of quantitative real-time PCR (qRT-PCR).

Supplementary Table 2 Components of quantitative real-time PCR (qRT-PCR) amplifications.

Supplementary Table 3 Primer sequences of quantitative real-time PCR (qRT-PCR).

Supplementary Table 4 Primer sequences of quantitative real-time PCR (PCR).

Supplementary Table 5 Components of PCR amplifications.

Supplementary Table 6 Primer sequences of quantitative real-time PCR (qRT-PCR).

Supplementary Table 7 Statistics analysis of RNA sequencing data from *Fusarium oxysporum*-treated and wild type (WT) Pdpap.

Supplementary Table 8 Differential expression gene statistics.

Supplementary Table 9 Read counts, fragments per kilobase million (FPKM), and descriptions of differentially expressed genes (DGEs).

Supplementary Table 10 Differential expressed *PdpapWRKYs* under *Fusarium oxysporum* treatment.

Supplementary Table 11 Differential expressed genes with more than 3-fold upregulated.
