# Supplementary figures and images for: Transcriptome Analysis Reveals the Important Role of WRKY28 in Fusarium oxysporum Resistance
Source: Front Plant Sci. 2021 Aug 20;12:720679. doi: 10.3389/fpls.2021.720679 (PMC8418079; doi:10.3389/fpls.2021.720679)

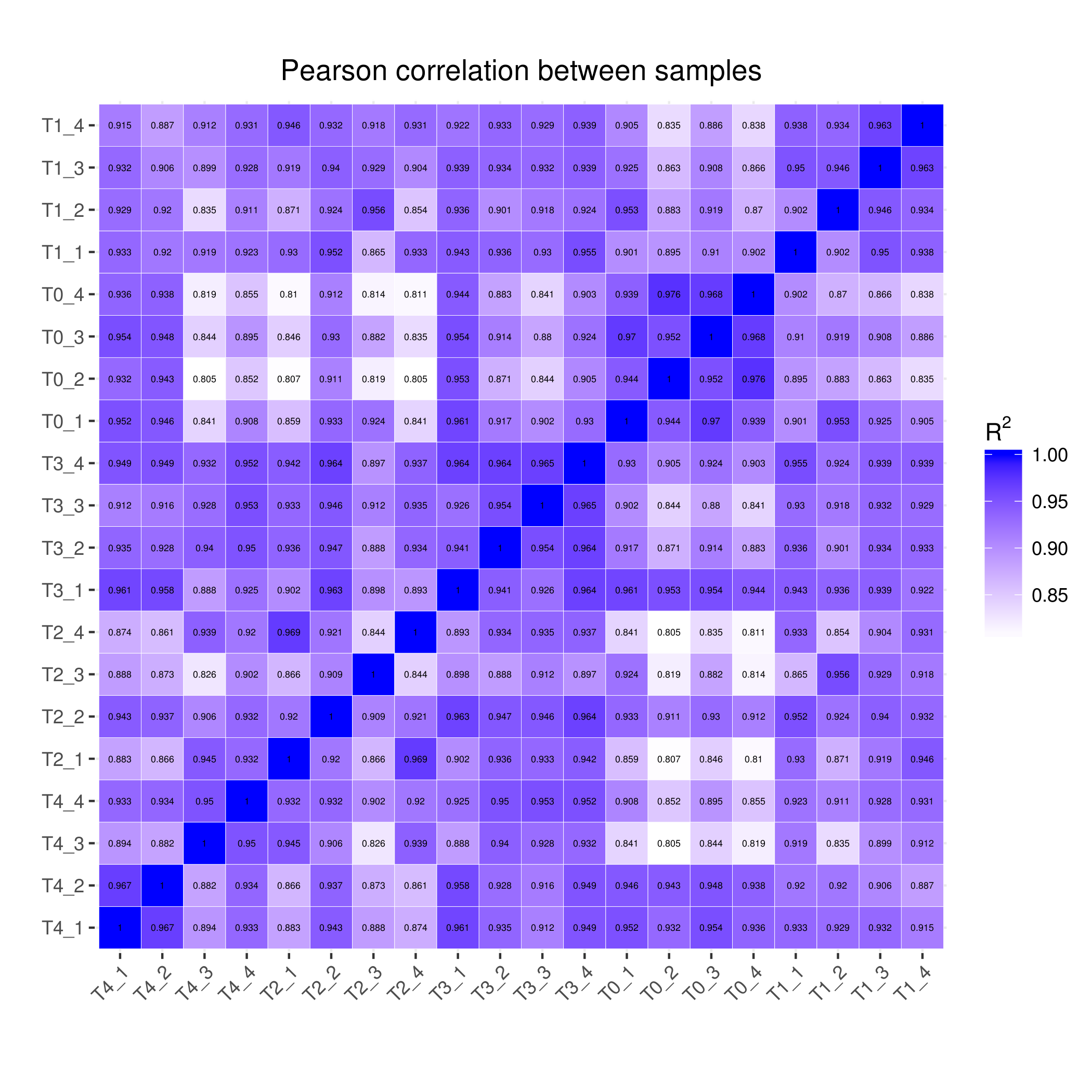

Supplement: Supplementary Figure 1 — General transcription patterns of all samples. Pearson correlation between Fusarium oxysporum-treated and wild type (WT) Populus davidiana × P. alba var. pyramidalis Louche (Pdpap). T0, T1, T2, T3, and T4 stand for the F. oxysporum-treated Pdpap by 0, 6, 12, 24, and 48 h, respectively. The numbers 1, 2, 3, and 4 after the treatment name stand for the four biological repetitions of the same treatment operation. [file Image_1.PNG]

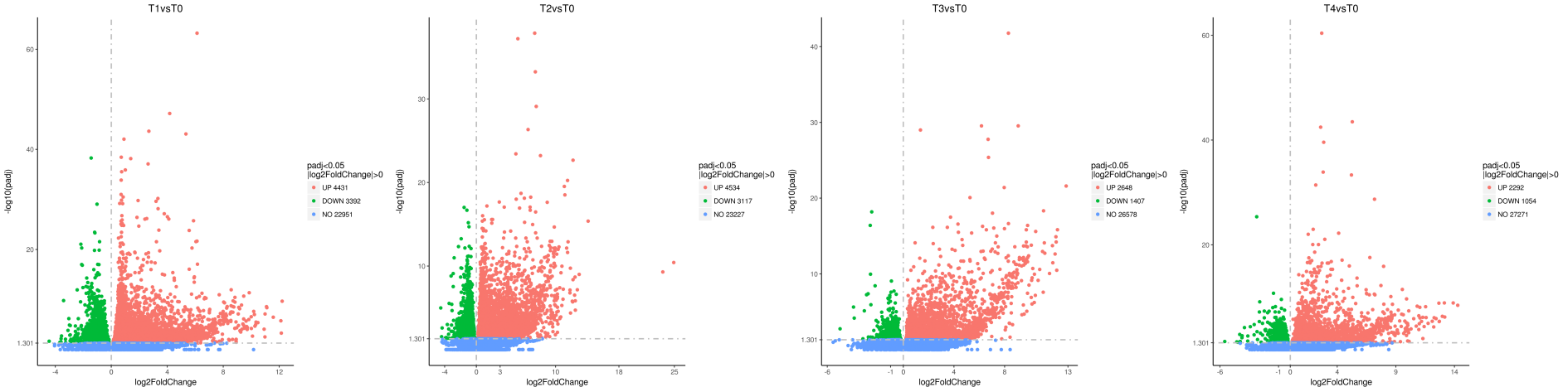

Supplement: Supplementary Figure 2 — Differential gene volcano map. T0, T1, T2, T3, and T4 stands for the F. oxysporum-treated Populus davidiana × P. alba var. pyramidalis Louche (Pdpap) by 0, 6, 12, 24, and 48 h, respectively. The numbers 1, 2, 3, and 4 after the treatment name stand for the four biological repetitions of the same treatment operation. [file Image_2.PNG]

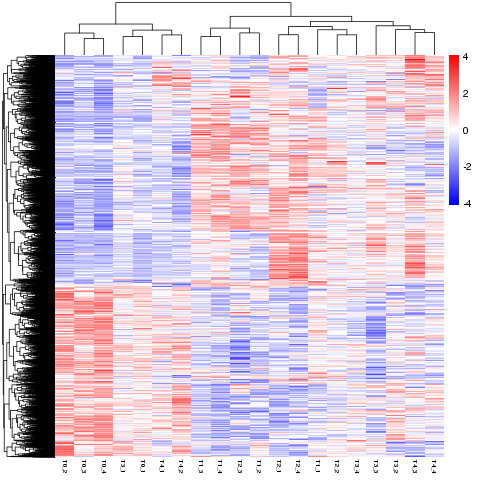

Supplement: Supplementary Figure 3 — Heat map of all the differentially expressed genes (DEGs) between the Fusarium oxysporum-treated and wild type (WT) groups of Populus davidiana × P. alba var. pyramidalis Louche (Pdpap). T0, T1, T2, T3, and T4 stand for the F. oxysporum-treated Pdpap by 0, 6, 12, 24, and 48 h, respectively. The numbers 1, 2, 3, and 4 after the treatment name stand for the 4 biological repetitions of the same treatment operation. [file Image_3.PNG]

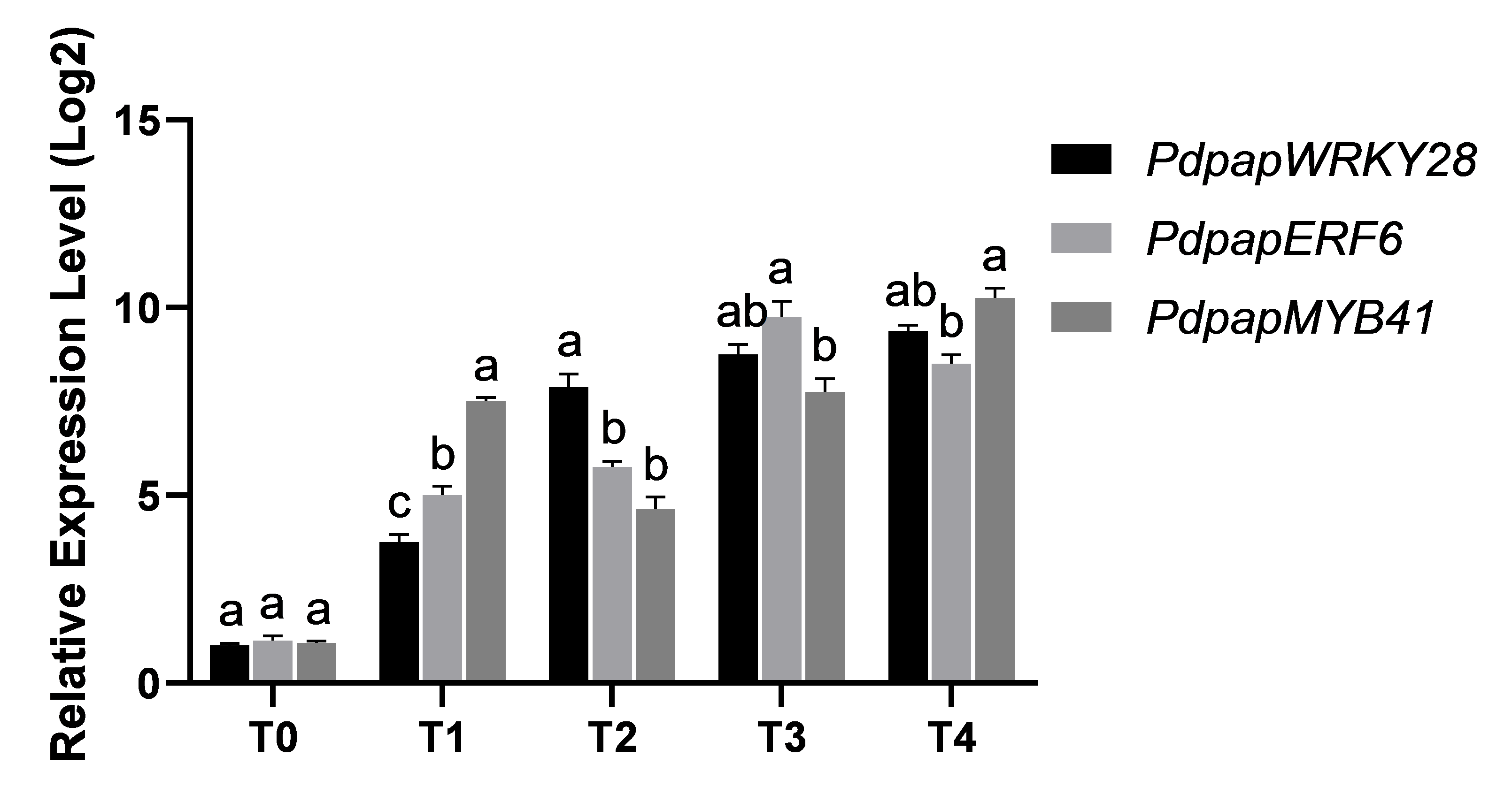

Supplement: Supplementary Figure 4 — Expression patterns analysis of candidate genes. The relative expression levels are obtained from the quantitative real-time PCR (qRT-PCR) measurements of Populus davidiana × P. alba var. pyramidalis Louche (Pdpap). Significant differences (P < 0.05) were indicated by different lowercase letters. Error bars represented the standard deviation of the three independent replicates. [file Image_4.TIF]

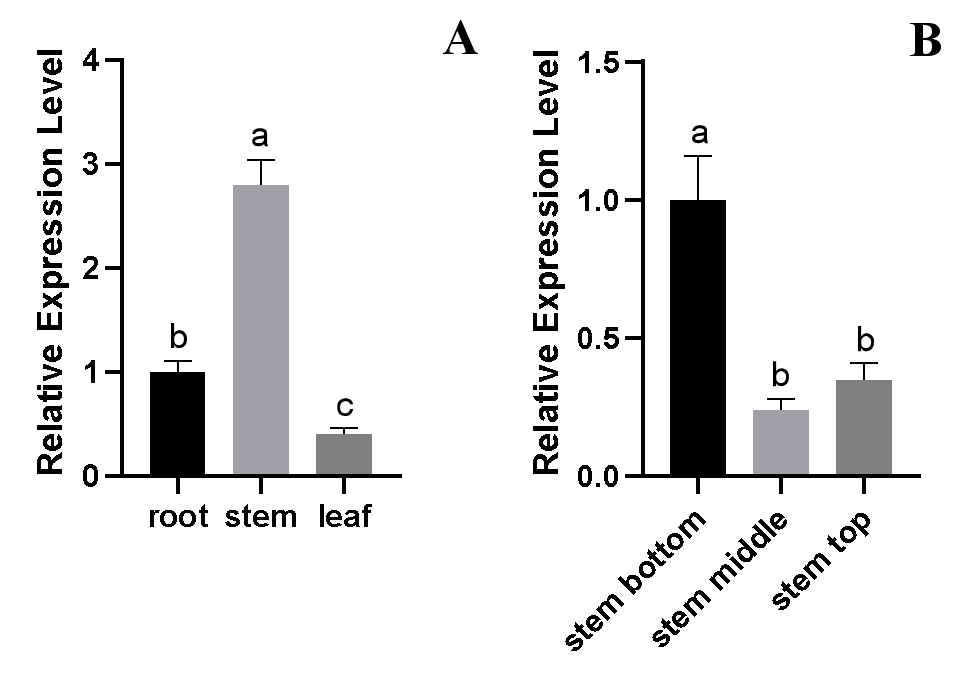

Supplement: Supplementary Figure 5 — Tissue-specific differential expression of the PdPapWRKY28 gene. (A) In different tissues of Populus davidiana × P. alba var. pyramidalis Louche (Pdpap); (B) In different parts of stems. The relative expression levels were obtained from the quantitative real-time PCR (qRT-PCR) measurements of Pdpap. Significant differences (P < 0.05) were indicated by different lowercase letters. Error bars represented the standard deviation of the three independent replicates. [file Image_5.TIF]

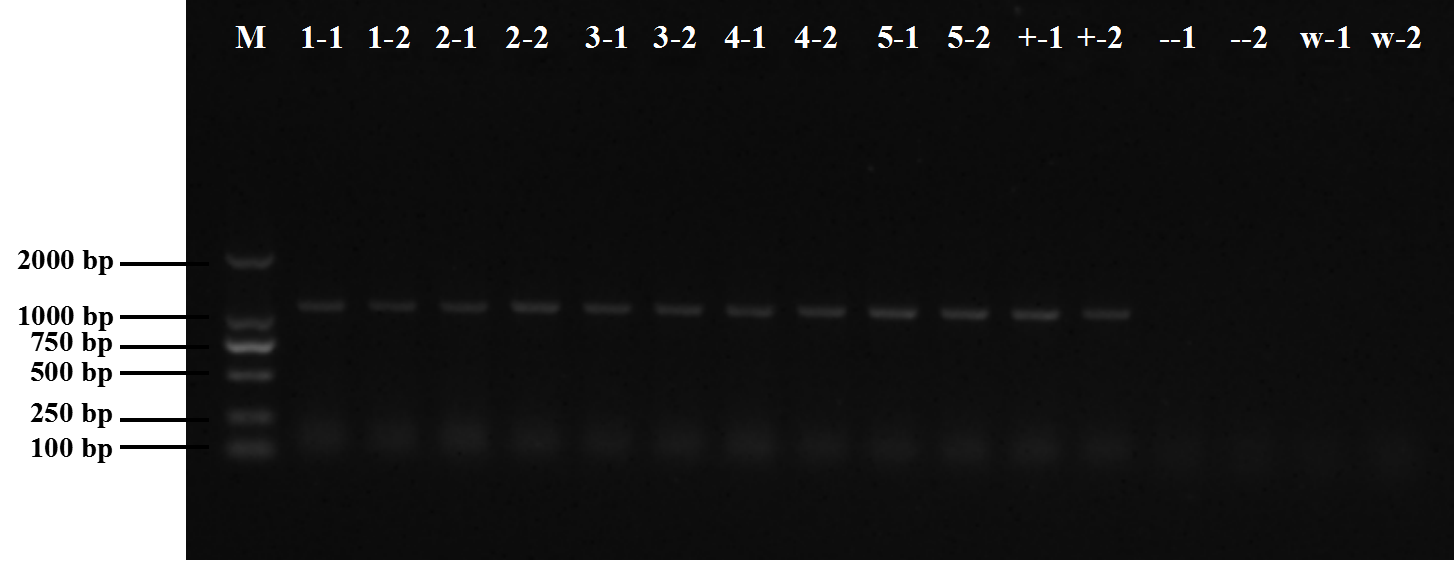

Supplement: Supplementary Figure 6 — Molecular detection in the putative transformants Populus davidiana × P. alba var. pyramidalis Louche (Pdpap) of PdPapWRKY28. M: DL2,000 Marker; 1–5: Five overexpressing putative transformant lines were used as amplification templates; +: Positive control using pBI121-PdPapWRKY28 plasmid as template; -, Negative control with water as template; W, Negative control with WT Pdpap as template;−1, PCR detection of putative transformant lines using pBI121-F and PdPapWRKY28-R as primers;−2, PCR detection of putative transformant lines with PdPapWRKY28-F and pBI121-R as primers. [file Image_6.TIF]
