# Supplementary material for: Transcriptome Analysis Reveals the Important Role of WRKY28 in Fusarium oxysporum Resistance
Source: Front Plant Sci. 2021 Aug 20;12:720679. doi: 10.3389/fpls.2021.720679 (PMC8418079; doi:10.3389/fpls.2021.720679)
Supplement: Supplementary Table 1 — Primer sequences of quantitative real-time PCR (qRT-PCR). [file Table_1.DOCX]

**Table S1**. Primer Sequences of qRT-PCR

| **Gene** | **Forward Primers (5'-3')** | **Reverse Primers (5'-3')** |
| --- | --- | --- |
| *Pdpapactin* | GCTGAGAGATTCCGTTGCCCTG | GGCGGTGATCTCCTTGCTCATT |
| *PdpapEF1-α* | TGGGTCGTGTTGAAACTGGTGT | GGCAGGATCGTCCTTGGAGTTC |
| 7458925 | TTGCTACGGGATGTGTAATGG | CTGATAACCCAATTGTGCCAG |
| 7469823 | CTCCAGCTAGTGGCAGTG | AGAAAATCCTCCACAGCTTCC |
| 7495696 | TTGCTACGGGATGTGTAATGG | TGATAACCCAGTTGTGCCAG |
| 7468053 | CTCTCCTCTATCTCCAAAACCAAA | CACGTCCATTATGACCCCG |
| 7489422 | GCAGATTACAGCGACAACG | TGCTCTTTCCCACTCATTACAG |
| 7464490 | GTGGAAGACAAAGTAGGGAAGG | ACCACTCCATGCCTCTTTG |
| 7465978 | TTCTCTTTCTCGCACCTCAC | GCAAACCCTAACGTCTATCTGG |
| 7478850 | TGATGTCTCCCAACTGAATGTG | TGATGTCTCCCAACTGAATGTG |
| 7461222 | AGGGACTAGGTTTGGGAGG | CGAAGATCTATTAGGGTGAACACG |
| 7494249 | GTACTCTCATGGTTCTAATAGTGGC | CAAAATCAATGGTTGGACCCC |
