# Supplementary material for: Transcriptome Analysis Reveals the Important Role of WRKY28 in Fusarium oxysporum Resistance
Source: Front Plant Sci. 2021 Aug 20;12:720679. doi: 10.3389/fpls.2021.720679 (PMC8418079; doi:10.3389/fpls.2021.720679)
Supplement: Supplementary Table 2 — Components of quantitative real-time PCR (qRT-PCR) amplifications. [file Table_2.DOCX]

**Table S2**. Components of qRT-PCR amplifications

| **Reation solution** | **Volume（μL）** |
| --- | --- |
| SYBR Green Realtime PCR Master Mix | 10 |
| cDNA | 2 |
| Primer F（10 μM） | 1 |
| Primer R（10 μM） | 1 |
| dd H_2_O | 6 |
