# Supplementary material for: Transcriptome Analysis Reveals the Important Role of WRKY28 in Fusarium oxysporum Resistance
Source: Front Plant Sci. 2021 Aug 20;12:720679. doi: 10.3389/fpls.2021.720679 (PMC8418079; doi:10.3389/fpls.2021.720679)
Supplement: Supplementary Table 4 — Primer sequences of quantitative real-time PCR (PCR). [file Table_4.DOC]

**Table S4** Primer sequences of PCR

| **The name of primers** | **Sequence** |
| --- | --- |
| pBI121-F | CAATCCCACTATCCTTCGC |
| pBI121-R | GTCCAGCTCGACCAGGAT |
| *PdPapWRKY28*-F | ACCTCCTCCTCTTTATCTAT |
| *PdPapWRKY28*-R | TACACTGAGAGAGACCCTTC |
