# Supplementary material for: Transcriptome Analysis Reveals the Important Role of WRKY28 in Fusarium oxysporum Resistance
Source: Front Plant Sci. 2021 Aug 20;12:720679. doi: 10.3389/fpls.2021.720679 (PMC8418079; doi:10.3389/fpls.2021.720679)
Supplement: Supplementary Table 5 — Components of PCR amplifications. [file Table_5.DOC]

Table S5 Components of PCR amplifications

| **Reation solution** | **Volume（μL）** |
| --- | --- |
| 10×Ex Taq PCR Buffer | 2 |
| dNTP Mix（10 mM） | 0.4 |
| Primer-F（10 μM） | 0.5 |
| Primer-R（10 μM） | 0.5 |
| Ex Taq（5 U/μL） | 0.3 |
| cDNA | 1 |
| ddH_2_O | 20.3 |
