# Supplementary material for: Transcriptome Analysis Reveals the Important Role of WRKY28 in Fusarium oxysporum Resistance
Source: Front Plant Sci. 2021 Aug 20;12:720679. doi: 10.3389/fpls.2021.720679 (PMC8418079; doi:10.3389/fpls.2021.720679)
Supplement: Supplementary Table 6 — Primer sequences of quantitative real-time PCR (qRT-PCR). [file Table_6.DOC]

**Table S6**. Primer Sequences of qRT-PCR

| **Gene** | **Forward Primers (5'-3')** | **Reverse Primers (5'-3')** |
| --- | --- | --- |
| *Pdpapactin* | GCTGAGAGATTCCGTTGCCCTG | GGCGGTGATCTCCTTGCTCATT |
| *PdpapEF1-α* | TGGGTCGTGTTGAAACTGGTGT | GGCAGGATCGTCCTTGGAGTTC |
| *NPR1* | GGCCGACGATACTTCCCTAGTT | TGCCCTCATAGTTTCCTGAGCT |
| *PR1* | ACACCACCGTGCAAGCCTATG | CGAGCAGAGTTACGCCAAACCA |
| *JAR1* | AGTGGTGAACCAAGCGAGGAG | GGATTTGCTGCACCTTGCTGTT |
| *COI* | GAGGTACTTGTGGGTGCATGGT | ACGCAGAACCTACCCTCGCT |
| *JAZ* | GGAAGCTCCATTGGCACAGATG | CCGGAGTTGGGTTGTTTGTCTG |
| *ORCA3* | AGAGGAGTGAGGCAAAGACCCT | TCCGGCTCTCGACGCTTAGT |
