# Supplementary material for: Transcriptome Analysis Reveals the Important Role of WRKY28 in Fusarium oxysporum Resistance
Source: Front Plant Sci. 2021 Aug 20;12:720679. doi: 10.3389/fpls.2021.720679 (PMC8418079; doi:10.3389/fpls.2021.720679)
Supplement: Supplementary Table 7 — Statistics analysis of RNA sequencing data from Fusarium oxysporum-treated and wild type (WT) Pdpap. [file Table_7.DOC]

**Table S7.** Statistics analysis of RNA sequencing data from *F. oxysporum*-treated and WT Pdpap.

| **Samples** | **Raw reads** | **Clean reads (%)** | **Clean bases (G)** | **Error rate (%)** | **Q20 (%)** | **Q30 (%)** | **GC (%)** | **Mapping rate (%)** |
| --- | --- | --- | --- | --- | --- | --- | --- | --- |
| T0_1 | 48,691,100 | 47,332,260 (97.21%) | 7.10 | 0.03 | 96.31 | 90.52 | 44.46 | 72.45 |
| T0_2 | 52,672,650 | 51,346,184 (97.48%) | 7.70 | 0.03 | 96.23 | 90.37 | 44.45 | 72.55 |
| T0_3 | 47,151,662 | 46,295,124 (98.18%) | 6.94 | 0.03 | 96.39 | 90.68 | 44.34 | 72.54 |
| T0_4 | 45,633,072 | 44,626,056 (97.79%) | 6.69 | 0.03 | 96.30 | 90.47 | 44.28 | 72.35 |
| T1_1 | 50,526,794 | 49,097,616 (97.17%) | 7.36 | 0.03 | 96.00 | 89.81 | 44.30 | 71.81 |
| T1_2 | 39,665,700 | 38,639,398 (97.41%) | 5.80 | 0.03 | 95.89 | 89.60 | 44.64 | 71.67 |
| T1_3 | 51,905,296 | 50,294,634 (96.90%) | 7.54 | 0.03 | 95.96 | 89.85 | 44.31 | 70.75 |
| T1_4 | 50,412,052 | 47,551,768 (94.33%) | 7.13 | 0.03 | 96.23 | 90.38 | 44.25 | 71.56 |
| T2_1 | 56,417,918 | 54,699,048 (96.95%) | 8.20 | 0.03 | 96.29 | 90.47 | 44.34 | 71.65 |
| T2_2 | 44,249,222 | 43,245,254 (97.73%) | 6.49 | 0.03 | 95.99 | 89.76 | 44.38 | 71.42 |
| T2_3 | 44,957,556 | 44,107,880 (98.11%) | 6.62 | 0.03 | 95.91 | 89.61 | 44.37 | 70.65 |
| T2_4 | 43,561,458 | 42,378,808 (97.29%) | 6.36 | 0.03 | 95.92 | 89.66 | 44.43 | 70.51 |
| T3_1 | 50,720,930 | 49,426,102 (97.45%) | 7.41 | 0.03 | 96.39 | 90.65 | 44.61 | 72.46 |
| T3_2 | 55,508,306 | 54,333,236 (97.88%) | 8.15 | 0.03 | 96.26 | 90.43 | 44.40 | 70.72 |
| T3_3 | 50,885,822 | 49,607,376 (97.49%) | 7.44 | 0.03 | 96.34 | 90.58 | 44.99 | 70.55 |
| T3_4 | 49,640,518 | 48,379,736 (97.46%) | 7.26 | 0.03 | 96.19 | 90.26 | 44.59 | 71.04 |
| T4_1 | 59,056,384 | 57,696,030 (97.70%) | 8.65 | 0.03 | 96.09 | 90.09 | 44.49 | 71.87 |
| T4_2 | 48,924,004 | 47,606,428 (97.31%) | 7.14 | 0.03 | 96.43 | 90.75 | 44.79 | 72.32 |
| T4_3 | 53,715,550 | 52,210,428 (97.20%) | 7.83 | 0.03 | 96.08 | 90.10 | 44.54 | 69.28 |
| T4_4 | 46,262,360 | 44,185,842 (95.51%) | 6.63 | 0.03 | 95.88 | 89.70 | 44.53 | 70.66 |
| **Total** | 990,558,354 | 963,059,208 | 144.44 |  |  |  |  |  |

Notes: T0, T1, T2, T3 and T4 stands for the *F. oxysporum*-treated Pdpap by 0, 6, 12, 24 and 48 h. 1, 2, 3 and 4 after the treatment name stands for 4 biological repetitions of the same treatment operation. Raw reads stands for the number of reads in the raw data. Clean reads stands for the number of reads after filtering the original data. Clean bases stands for the number of bases after filtering the original data. Error rate stands for the error rate of the overall data in the sequencing process. Q20 stands for the percentage of bases with a Phred value >20 in the total bases, where Phred=-10log10(e). Q30 stands for the percentage of bases with a Phred value >30 in the total bases, where Phred=-10log10(e). GC stands for the percentage of G and C in the four bases among clean reads. Mapping rate stands for the ratio of the reads number compared to the genome database accounting to the number of clean reads.
