# Supplementary material for: Transcriptome Analysis Reveals the Important Role of WRKY28 in Fusarium oxysporum Resistance
Source: Front Plant Sci. 2021 Aug 20;12:720679. doi: 10.3389/fpls.2021.720679 (PMC8418079; doi:10.3389/fpls.2021.720679)
Supplement: Supplementary Table 8 — Differential expression gene statistics. [file Table_8.DOC]

**Table S8** Differential expression gene statistics

| **compare** | **all** | **up** | **down** | **threshold** |
| --- | --- | --- | --- | --- |
| T1vsT0 | 7,823 | 4,431 | 3,392 | DESeq2 *P* adj<0.05 |log_2_FoldChange|>0 |
| T2vsT0 | 7,651 | 4,534 | 3,117 |
| T3vsT0 | 4,055 | 2,648 | 1,407 |
| T4vsT0 | 3,346 | 2,292 | 1,054 |

Notes: All stands for the total number of differential genes in the comparison combination. Up stands for the number of up-regulated differential genes in the comparison combination. Down stands for the number of down-regulated differential genes in the comparison combination. Threshold stands for the software and threshold for differential gene screening in the comparison combination. T0, T1, T2, T3 and T4 stands for the *F. oxysporum*-treated Pdpap by 0, 6, 12, 24 and 48 h. 1, 2, 3 and 4 after the treatment name stands for 4 biological repetitions of the same treatment operation.
