# Supplementary material for: Transcriptome Analysis Reveals the Important Role of WRKY28 in Fusarium oxysporum Resistance
Source: Front Plant Sci. 2021 Aug 20;12:720679. doi: 10.3389/fpls.2021.720679 (PMC8418079; doi:10.3389/fpls.2021.720679)
Supplement: Supplementary Table 11 — Differential expressed genes with more than 3-fold upregulated. [file Table_11.DOC]

**Table S11** Differential expressed genes with more than 3 fold up-regulated

| **Gene ID** | **log_2_FoldChange** | | | | **Description** |
| --- | --- | --- | --- | --- | --- |
|  | **T1vsT0** | **T2vsT0** | **T3vsT0** | **T4vsT0** |  |
| 7493352 | 5.44 | 3.86 | 4.86 | 6.95 | EG45-like domain containing protein |
| 7461382 | 6.46 | 8.96 | 9.81 | 10.96 | peroxidase 15 |
| 18097308 | 12.12 | 11.82 | 12.17 | 14.28 | miraculin |
| 18103511 | 9.83 | 11.40 | 11.98 | 13.14 | berberine bridge enzyme-like 28 |
| 7498090 | 6.34 | 4.43 | 4.64 | 6.46 | basic endochitinase CHB4 |
| 18108499 | 4.11 | 3.29 | 3.43 | 3.66 | endochitinase EP3 |
| 7489112 | 9.63 | 9.73 | 10.13 | 13.17 | cannabidiolic acid synthase-like 1 |
| 18104080 | 8.68 | 6.34 | 7.38 | 9.64 | pathogenesis-related protein PR-4 |
| 18098581 | 7.96 | 11.51 | 11.54 | 11.76 | hyoscyamine 6-dioxygenase |
| 7485050 | 12.15 | 9.50 | 10.35 | 10.96 | kunitz trypsin inhibitor 1 |
| 7485051 | 10.09 | 9.63 | 10.11 | 12.43 | alpha-amylase/subtilisin inhibitor |
| 7468989 | 4.74 | 7.07 | 8.30 | 9.94 | glucan endo-1%2C3-beta-glucosidase%2C basic isoform |
| 7489699 | 4.10 | 5.14 | 5.93 | 6.02 | GDSL esterase/lipase 1 |
| 7485052 | 11.58 | 9.04 | 9.15 | 9.79 | endogenous alpha-amylase/subtilisin inhibitor |
| 112326219 | 7.75 | 4.13 | 5.59 | 8.54 | - |
| 18100635 | 5.64 | 8.08 | 9.89 | 11.19 | uncharacterized LOC18100635 |
| 7489102 | 8.04 | 9.30 | 9.71 | 10.82 | berberine bridge enzyme-like 17 |
| 18108092 | 10.00 | 9.43 | 9.51 | 9.84 | alpha-amylase/subtilisin inhibitor |
| 18103617 | 10.34 | 7.98 | 8.84 | 10.10 | acidic endochitinase SE2 |
| 18097749 | 5.19 | 4.66 | 4.77 | 5.04 | BURP domain protein RD22 |
| 7460508 | 4.46 | 3.97 | 4.25 | 4.82 | pleiotropic drug resistance protein 1%2C transcript variant X1 |
| 7471680 | 6.22 | 8.34 | 8.65 | 9.85 | berberine bridge enzyme-like 18 |
| 7489736 | 6.13 | 3.94 | 4.11 | 4.13 | mitogen-activated protein kinase kinase kinase 17 |
| 7460016 | 4.38 | 5.64 | 6.41 | 7.36 | probable glutathione S-transferase |
| 7480395 | 5.71 | 8.07 | 8.26 | 9.14 | berberine bridge enzyme-like 18 |
| 112324441 | 5.47 | 7.39 | 8.08 | 9.20 | uncharacterized LOC112324441 |
| 18103834 | 5.94 | 6.87 | 7.97 | 9.14 | uncharacterized LOC18103834 |
| 7482570 | 5.00 | 6.22 | 7.89 | 8.01 | peroxidase 2 |
| 18095297 | 3.01 | 3.28 | 3.30 | 3.34 | probable WRKY transcription factor 28 |
| 7494517 | 4.49 | 5.16 | 5.88 | 6.91 | universal stress protein PHOS32%2C transcript variant X1 |
| 7471649 | 4.82 | 4.14 | 4.40 | 4.57 | ethylene-responsive transcription factor 6 |
| 7454469 | 5.80 | 5.16 | 5.70 | 7.44 | dehydration-responsive element-binding protein 1A |
| 18104817 | 5.05 | 5.16 | 5.20 | 6.15 | cytochrome P450 81F3 |
| 18095667 | 5.23 | 5.43 | 5.48 | 5.63 | transcription factor MYB41 |
| 7490007 | 5.21 | 5.94 | 6.25 | 6.30 | peroxidase 10 |
| 7467473 | 5.71 | 4.87 | 5.98 | 6.26 | endochitinase EP3 |
